# Genetic pathways regulating the longitudinal acquisition of cocaine self-administration in a panel of inbred and recombinant inbred mice

**DOI:** 10.1016/j.celrep.2023.112856

**Published:** 2023-07-22

**Authors:** Arshad H. Khan, Jared R. Bagley, Nathan LaPierre, Carlos Gonzalez-Figueroa, Tadeo C. Spencer, Mudra Choudhury, Xinshu Xiao, Eleazar Eskin, James D. Jentsch, Desmond J. Smith

**Affiliations:** 1 Department of Molecular and Medical Pharmacology, Geffen School of Medicine, UCLA, Los Angeles, CA 90095, USA; 2 Department of Psychology, Binghamton University, Binghamton, NY, USA; 3 Department of Computer Science, UCLA, Los Angeles, CA 90095, USA; 4 Department of Integrative Biology and Physiology, UCLA, Los Angeles, CA 90095, USA; 5 Department of Computational Medicine, UCLA, Los Angeles, CA 90095, USA; 6 Present address: Cedars-Sinai Medical Center, 8700 Beverly Blvd, Los Angeles, CA 90048, USA; 7 Present address: Department of Human Genetics, University of Chicago, Chicago, IL 60637, USA; 8 Lead contact

## Abstract

To identify addiction genes, we evaluate intravenous self-administration of cocaine or saline in 84 inbred and recombinant inbred mouse strains over 10 days. We integrate the behavior data with brain RNA-seq data from 41 strains. The self-administration of cocaine and that of saline are genetically distinct. We maximize power to map loci for cocaine intake by using a linear mixed model to account for this longitudinal phenotype while correcting for population structure. A total of 15 unique significant loci are identified in the genome-wide association study. A transcriptome-wide association study highlights the *Trpv2* ion channel as a key locus for cocaine self-administration as well as identifying 17 additional genes, including *Arhgef26, Slc18b1*, and *Slco5a1*. We find numerous instances where alternate splice site selection or RNA editing altered transcript abundance. Our work emphasizes the importance of *Trpv2*, an ionotropic cannabinoid receptor, for the response to cocaine.

## In brief

Khan et al. use a panel of 84 inbred and recombinant inbred mouse strains to identify genes for cocaine self-administration. By combining genetic mapping data with RNA sequencing data from the brain of 41 strains, increased expression of the ionotropic cannabinoid receptor *Trpv2* is linked to decreased cocaine use.

## Graphical Abstract

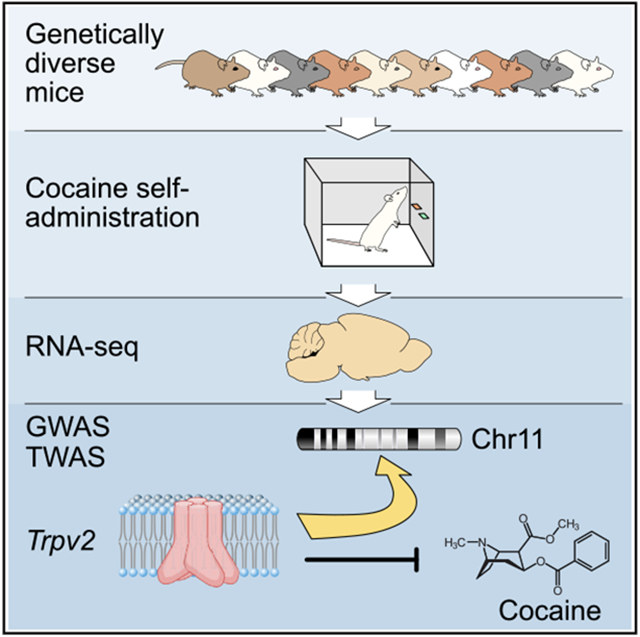

## INTRODUCTION

Cocaine use disorders are a significant health burden. In the United States, 2 million people use cocaine once a month or more, and greater than 850,000 individuals are dependent on the drug.^1-4^ Deaths due to cocaine overdose in 2018 were 4.5 per 100,000 standard population.^5^

Cocaine acts by blocking the reuptake transporters for dopamine, serotonin, and norepinephrine in presynaptic nerve terminals, thus increasing the concentrations of these neurotransmitters in the synaptic cleft. The rewarding effects of cocaine are largely mediated by increased dopaminergic neurotransmission in the limbic system, in particular the nucleus accumbens (NAc) and prefrontal cortex.^2,4^

Addiction to cocaine is a complex trait, with multiple environmental and genetic factors. The broad sense heritability (H^2^) for cocaine use in humans is ~0.32–0.79, and the additive heritability (h^2^) for cocaine dependence is ~0.27–0.30. There is evidence of overlap between the genetic risk factors for cocaine use and other addictive drugs, in particular cannabis.^6^

Properly powered genome-wide association studies (GWASs) of cocaine addiction in humans await ascertainment of adequate population sizes, likely tens to hundreds of thousands of individuals. Hurdles in obtaining sufficient numbers include difficulties in recruiting cocaine-dependent individuals, gene-environment interactions, and population and phenotypic heterogeneity. One genome-wide significant variant for cocaine use disorder has been identified in the *FAM53B* gene.^7^ Analysis of the same data using a gene-based test identified *C1QL2, STK38, KCTD20*, and *NDUFB9* as addiction genes, while a meta-analysis identified *HIST1H2BD*.^8-10^ A recent GWAS employed gene-environment interactions to identify 13 significant genes, and another investigation found two significant loci associated with the latency from cocaine use to dependence.^11,12^

Genetic studies in mice can provide useful insights into cocaine addiction, since control of environment and behavioral endpoints is easily obtained. One investigation evaluated cocaine self-administration in 39 strains of recombinant inbred BXD mice. A cocaine self-administration quantitative trait locus (QTL) was found to harbor a *trans* expression QTL (eQTL) for *Fam53b*.^13^ Impulsivity and other behavioral endophenotypes may be valuable surrogates for genetic mapping of cocaine use disorders in rodent models.^14-17^ For example, one study using BXD mice showed that poor reversal learning, which indicates a lack of inhibitory control, was associated with greater cocaine self-administration.^18^

Further evidence for a genetic basis of cocaine use was the observation that an acute dose of the drug caused significant differences in locomotor activity across 45 inbred mouse strains.^19^ Divergence in sensitization to cocaine was also found using 51 genetically diverse collaborative cross strains and their inbred founders.^20^ An elegant study using two closely related substrains of the C57BL/6 mouse strain revealed that a missense mutation in *Cyfip2* results in altered sensitization to cocaine.^21^

The hybrid mouse diversity panel (HMDP) is a collection of ~30 inbred and ~70 recombinant inbred mouse strains that can be used for association mapping of complex traits, including behavior.^22,22-24^ The inbred strains have a large number of recombination events facilitating high resolution genetic mapping, while the recombinant inbred strains increase statistical power. Because the HMDP is genetically stable, it is possible to layer multiple phenotypes on the panel, providing ever more powerful insights.

We have previously used the HMDP to evaluate cocaine and saline intravenous self-administration (IVSA) over a 10-day testing period.^25^ The panel showed high phenotypic diversity in cocaine and saline IVSA, consistent with a genetic basis for these traits. Genetic mapping revealed significant loci for cocaine IVSA on chromosomes 3 and 14 and another suggestive locus on chromosome 3. Massively parallel RNA sequencing (RNA-seq) of the NAc and medial frontal cortex (mFC) provided *cis* eQTLs that could be employed to narrow down candidate genes for cocaine self-administration. However, the power of the study was not completely realized since the genome scans used five sequential sets of binned 2-day intervals, rather than exploiting the full longitudinal nature of the datasets.

In this study, we used a linear mixed model to analyze the same dataset and identify loci that affect longitudinal cocaine IVSA while correcting for population structure. We further extended the analyses of RNA-seq data by mapping *cis* and *trans* QTLs affecting transcript, spliceform, and editing abundance. Splicing and editing events that influenced transcript abundance were identified. We then used transcriptome-wide association studies (TWASs) to combine the results of the behavioral GWASs with the RNA-seq data, providing confirmatory support for genes underlying cocaine use while also suggesting additional genes.

## RESULTS

### Cocaine and saline self-administration

As described previously, we evaluated 84 strains of the HMDP for cocaine (479 mice) or saline IVSA (477 mice) over a 10-day testing period.^25^ Animals could press either of two levers in the testing chamber; one caused the infusate (cocaine or saline) to be delivered, and the other was inactive. A total of four behavioral endpoints were evaluated: number of infusions, active lever presses, percent active lever presses, and inactive lever presses (Table S1). Normalized data were used for all analyses.

### Behavioral covariates

To evaluate the influences on behavior independent of genetic background, we used a linear mixed model implemented in lme4 with fixed effects of testing chamber, active lever (left vs. right), age, sex, and testing day, along with a random effect of strain.^26^

Testing chamber was a significant effect on all behavioral endpoints for both cocaine (η^2^_p_ = 0.047 ± 0.009, χ^2^ = 227, degrees of freedom [df] = 45, p < 2.2 × 10^−16^, percent lever presses, least significant endpoints quoted) and saline (η^2^_p_ = 0.037 ± 0.009, χ^2^ = 164, df = 46, p = 3.2 × 10^−15^, percent active lever presses) (Figure S1A). The significant effect of testing chamber may reflect the fact that chambers were assigned non-randomly, to minimize the consequences of having multiple mice from the same strain in the same chamber.^25^

There was significantly higher self-administration of both cocaine (left vs. right = 0.19 ± 0.02, t[1, 4672] = 8.5, p < 2.2 × 10^−16^, Kenward-Roger df, active lever presses) and saline (left vs. right = 0.10 ± 0.02, t[1, 4650] = 4.9, p = 9.7 × 10^−7^, infusions) when the active lever was on the left compared to the right (Figure S1A). Conversely, inactive lever presses were significantly higher when the active lever was on the right(cocaine,leftvs. right = −0.43 ± 0.02, t[1, 4681] = 19.3, p< 2.2 × 10^−16^; saline, leftvs. right = −0.32 ± 0.02, t[1, 4652] = 14.8, p < 2.2 × 10^−16^). Even though significant, the relatively modest effect size of lever placement was similar for all covariates and reflected the large sample size.

Cocaine behavioral endpoints showed no significant effect of age (Figure S1A), while all saline measures decreased with age (coefficient = −0.05 ± 0.007, t[1, 4704] = 7.7, p = 1.4 × 10^−14^, infusions) except percent active lever presses, which was non-significant. Males had significantly higher measures for all cocaine endpoints (males vs. females = 0.07 ± 0.02, t[1, 4672] = 3.3, p = 1.2 × 10^−3^, infusions) except percent active lever presses, which was non-significant (Figure S1A). In contrast, sex had no significant effect on saline endpoints.

Mice working for cocaine compared to saline showed significantly higher infusions, active lever presses, and percent active lever presses (active lever presses, cocaine vs. saline = 0.12 ± 0.02, t[1, 9431] = 7.5, p = 9.1 × 10^−14^) (Figure S1B). In contrast, mice showed significantly higher inactive lever presses for saline than cocaine (cocaine vs. saline = −0.31 ± 0.02, t[1,9433] = 19.5, p < 2.2 × 10^−16^).

For cocaine over the 10 days of the experiment, there was a significant increase in percent lever presses (coefficient = 0.026 ± 0.004, t[1, 4657] = 6.2, p = 5.1 × 10^−10^) and a significant decrease in inactive lever presses (coefficient = −0.019 ± 0.004, t[1, 4657] = 5.2, p = 2.4 × 10^−7^) (Figure S1B). Saline showed the converse pattern, with a significant decrease in percent lever presses (coefficient = −0.029 ± 0.004, t[1, 4636] = 7.1, p = 1.1 × 10^−12^) and a significant increase in inactive lever presses (coefficient = 0.021 ± 0.004, t[1, 4636] = 6.2, p = 5.9 × 10^−10^). Infusions and active lever presses for either cocaine or saline showed no significant effect of experimental day.

### Differing genetic basis for cocaine and saline IVSA

**Figure 1.**
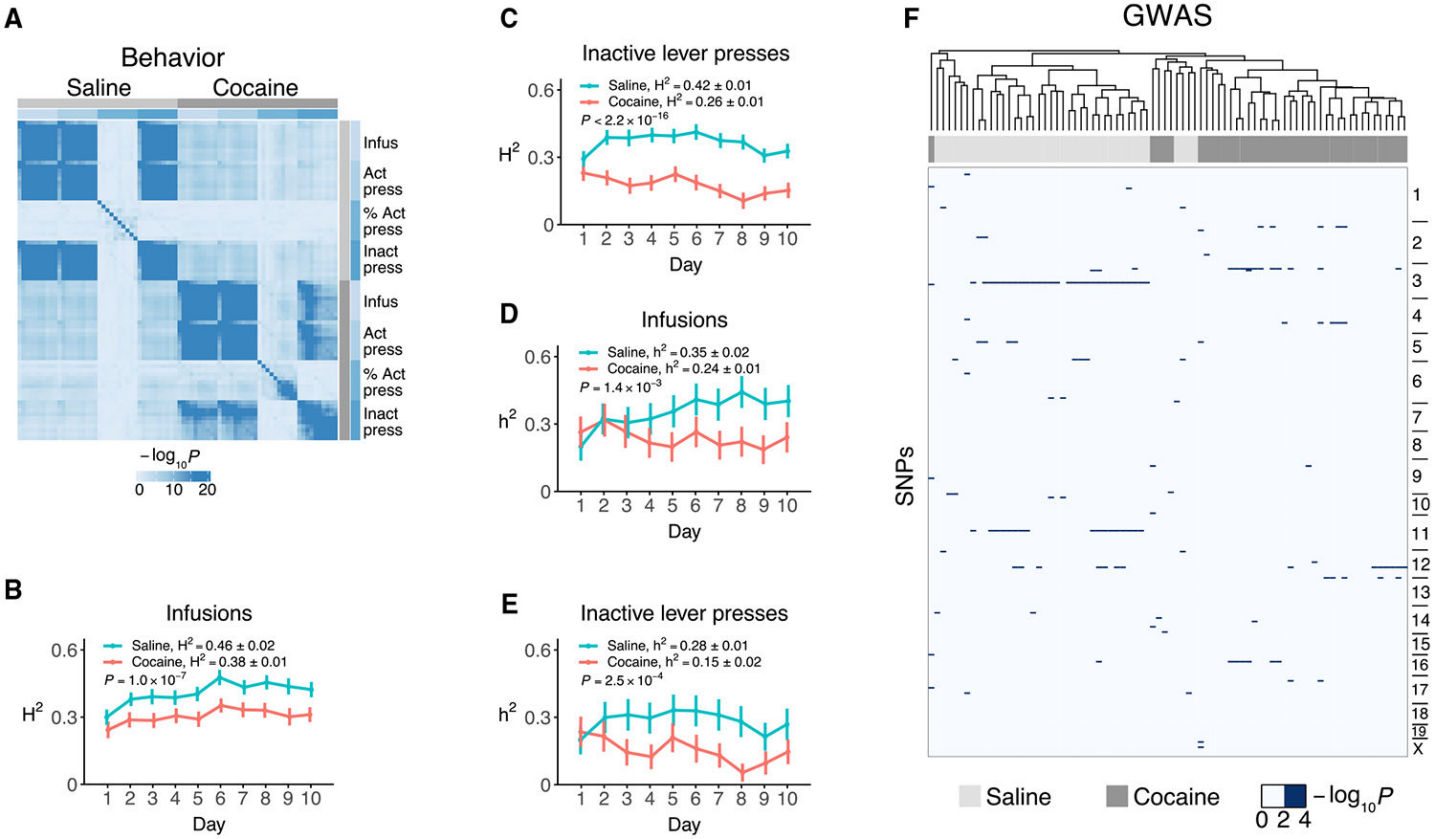
Cocaine and saline IVSA (A) Correlation significance values (−log_10_p) between behaviors averaged by strain. Each row and column represents a different day of testing. Gray key indicates saline or cocaine. Blue key indicates behavioral endpoint. (B) Broad sense heritability (H^2^) for cocaine and saline infusions over 10 days. p value compares cocaine and saline. Means ± SEM. (C) H^2^ for inactive lever presses. (D) Additive heritability (h^2^) for infusions. (E) h^2^ for inactive lever presses. (F) GWAS clustering. Columns represent GWAS for cocaine and saline IVSA on individual days for the four behavioral endpoints. Dendrogram shows unsupervised clustering of columns. Rows represent SNPs maintained in genome order; chromosomes are indicated. Color represents GWAS −log_10_p value. See also Figures S1 and S2 and Table S1.

A number of analyses suggested distinct genetics for cocaine and saline taking. We found significant correlations between infusates for behavioral measures averaged by strain (cocaine vs. saline, *R* = 0.31 ± 5.0 × 10^−3^), but within the same infusate, the correlations were significantly higher (combined cocaine vs. cocaine and saline vs. saline, *R* = 0.51 ± 9.3 × 10^−3^; comparison between and within infusates t = 18.8, df = 2,408, p < 2.2 × 10^−16^). This observation suggests different behavioral responses to the two regimens (Figures 1A, S1C, and S1D).

Over the 10-day period, all measures of self-administration showed significant H^2^, both for saline (e.g., infusion H^2^ = 0.46 ± 0.02, t[1, 9] = 29.4, p = 3.0 × 10^−10^, one sample t test) and cocaine (infusion H^2^ = 0.38 ± 0.01, t[1, 9] = 38.9, p = 2.5 × 10^−11^) (Figures 1B, 1C, and S2A-S2C). However, there was significantly higher H^2^ for saline compared to cocaine for infusions, active lever presses, and inactive lever presses (infusion, p = 1.0 × 10^−7^, sampling without replacement).

h^2^ was also significant for all measures of self-administration (saline infusion h^2^ = 0.35 ± 0.02, t[1, 9] = 16.1, p = 6.1 × 10^−8^; cocaine infusion h^2^ = 0.24 ± 0.01, t[1, 9] = 18.7, p = 1.7 × 10^−8^) (Figures 1D, 1E, and S2D-S2F). Similar to H^2^, h^2^ was significantly higher for saline than cocaine for infusions, active lever presses, and inactive lever presses (infusion, t[1, 9] = 4.6, p = 1.4 × 10^−3^). The aversive properties of cocaine may outweigh its reinforcing properties in mice, “masking” the action of genes that would otherwise contribute to self-administration. H^2^ and h^2^ were roughly consistent with estimates from human populations.

Cocaine inactive lever presses showed a significant decrease in h^2^ during the experiment (time coefficient = −1.3 × 10^−2^ ± 3.3 × 10^−3^, p = 2.0 × 10^−4^) (Figures 1E and S2D), while saline did not (time coefficient = −5.5 × 10^−4^ ± 4.2 × 10^−3^, p = 0.87). In fact, h^2^ for cocaine inactive lever presses on day 8 was non-significant (h^2^ = 0.05 ± 0.04, Z = 1.3, p = 0.18). The decrease in h^2^ for cocaine inactive lever presses occurred simultaneously with a switch to the active lever (Figure S1B), suggesting reduced additive variance as the cause of the decrease. Consistent with this reasoning, additive variance for cocaine on day 8 was non-significant (σ^2^ = 0.04 ± 0.02, Z = 1.8, p = 0.08), while environmental variance remained significant (σ^2^ = 0.62 ± 0.02, Z = 27.7, p < 2.2 × 10^−16^) (Figures S2D-S2F).

To further explore the genetic basis for cocaine and saline IVSA, we performed a separate GWAS for each of the 10 days using the four behavioral endpoints.^25^ We employed a linear mixed model using FaST-LMM software to correct for population structure.^27^ Although none of the GWASs for the individual days exceeded genome-wide significance, clustering of the results showed segregation of the genome scans for cocaine and saline (Figure 1F). Further, there was a significantly higher correlation of GWAS results within infusate (cocaine vs. cocaine and saline vs. saline; *R* = 0.35 ± 8.0 × 10^−3^) than between (cocaine vs. saline) (*R* = 0.10 ± 2.6 × 10^−3^, t[1, 1898] = 29.0, p < 2.2 × 10^−16^) (Figure S1D). Together, these observations indicate different genetic factors for cocaine and saline IVSA.

### A longitudinal analysis increases statistical power

**Figure 2.**
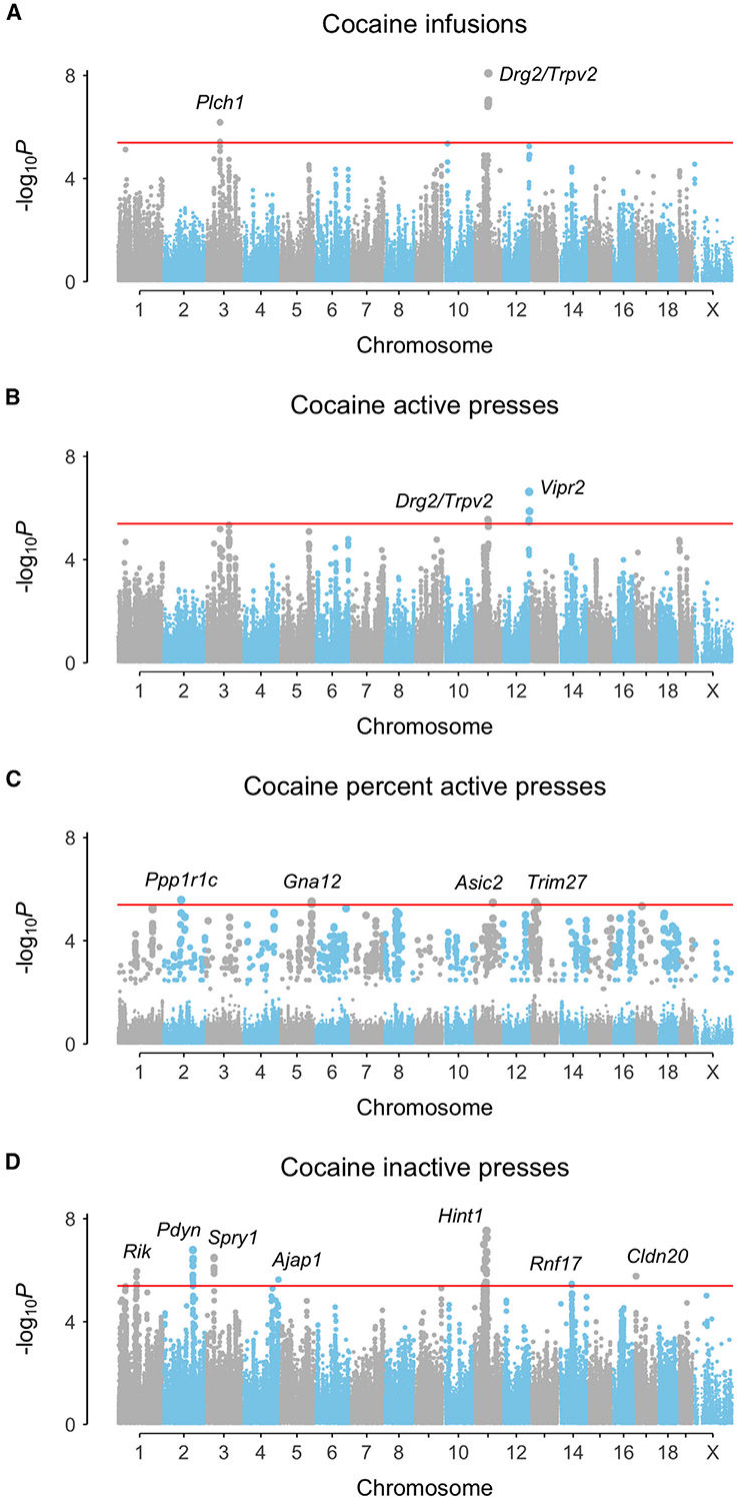
Genome scans of longitudinal cocaine IVSA phenotypes (A) Infusions. (B) Active presses. (C) Percent active presses. (D) Inactive presses. *Rik, A630001G21Rik*. Red horizontal line, significance threshold p < 4.1 × 10^−6^. See also Figure S3.

To improve statistical power, we evaluated the longitudinal behavioral phenotypes using a linear mixed model implemented in GMMAT software.^28^ Single-nucleotide polymorphisms (SNPs) were treated in the model as fixed effects on the normalized IVSA measures. The model further incorporated fixed and random slopes of testing day as a continuous variable plus fixed covariate effects of age, sex, active lever position (left or right), testing chamber, and cohort. Genetic relatedness was corrected via a random intercept derived from an SNP-based kinship matrix. A total of 17 significant loci were identified, of which 15 were unique (Figure 2, Table 1). The lack of intermediate −log_10_p values in the percent cocaine active lever presses (Figure 2C) may reflect the fact that 12% of mice pressed neither active nor inactive lever, rendering these data undefined.

We nominated plausible candidate genes for cocaine self-administration based on proximity to the behavioral loci and known roles in addiction, dopamine neurotransmission, or the brain. We gave higher priority to candidate genes that were also *cis* eQTLs or supported by TWASs. Of the 15 unique significant loci, eight were supported by evidence from *cis* eQTLs or TWASs (Table 1).

As expected, loci showed allelic differences in behavioral endpoints over the time course of the study (Figure S3A). Of the 17 loci for longitudinal cocaine IVSA, only one was also significant for longitudinal saline intake (rs30059671, inactive lever presses, *Spry1*, p = 1.8 × 10^−6^). Quantile-quantile plots for longitudinal cocaine IVSA showed deviations from normality (Figure S1E), reflecting longer linkage disequilibrium blocks in the HMDP compared to human, as well as the longitudinal nature of the phenotypes.^29^

### RNA-seq

To better discern genes for cocaine self-administration, RNA-seq was performed on NAc and mFC from 41 cocaine- and saline-exposed strains in the HMDP. NAc and mFC were chosen because the enhanced dopaminergic signaling in these brain regions caused by cocaine is responsible for much of the drug’s action.^2,4^ A total of 72.6 ± 1.1 M paired-end reads were obtained per strain in each brain region for cocaine and 73.0 ± 1.1 M for saline.^25^

Principal components analyses were performed using transcript abundance, splicing (percent spliced in, psi or ψ) and RNA editing (percent edited, phi or φ) (Figure S3B). Samples showed strong separation due to region, some separation due to batch, but little or no separation based on sex or infusate. A total of four samples showed possible misattribution based on region, corresponding to an error rate of 1%. This error rate is comparable to, or better than, other genome-scale studies.^30,31^ To avoid over-fitting, we elected not to correct the putatively mis-assigned samples.

### Gene regulation

Changes in transcript and isoform abundance may be caused directly by the infusate or indirectly influenced by genetic background. Our study is nearly balanced with respect to infusate and mouse strain, so population structure is unlikely to be an appreciable confound. Nevertheless, to ensure that we identified expression changes independent of genetic background, we used a linear mixed model implemented in lme4qtl to correct for population structure via a kinship matrix.^32^ The model also incorporated all known dependent variables, with fixed effects of brain region, sex, infusate, sex × infusate interaction, and RNA-seq batch, each assigned its own p value. We included only one interaction term, which evaluated sex-dependent effects of cocaine on gene expression. Additional interactions could exhaust the power of the model and lead to unacceptable false positive and false negative rates.^33^

### Genes regulated by cocaine

**Figure 3.**
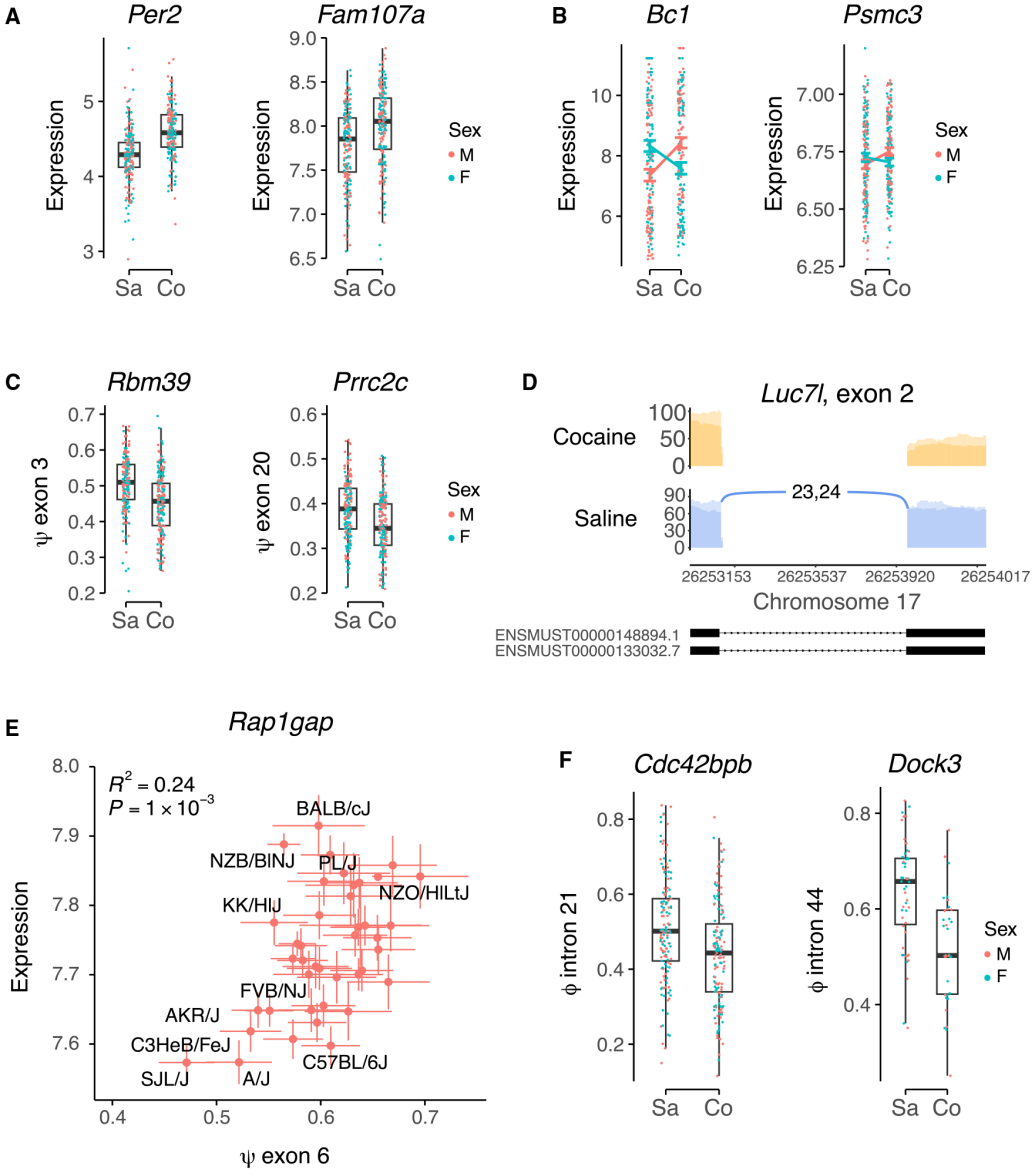
Regulation of gene expression by cocaine (A) Normalized transcript abundance. *Per2, Fam107a*, both FDR < 2.2 × 10^−16^. Sa, saline; Co, cocaine. M, male; F, female. (B) Sex × infusate interactions for transcript abundance. *Bc1*, FDR = 3.8 × 10^−12^; Psmc3, FDR = 3.5 × 10^−3^. Means ± SEM. (C) Splicing. *Rbm39*, exon 3, FDR = 2.2 × 10^−12^; *Prrc2c*, exon 20, FDR = 4.0 × 10^−6^. (D) Sashimi plot of cocaine-regulated splicing of *Luc7l*, exon 2. FDR = 7.6 × 10^−3^. (E) Alternate splicing of *Rap1gap*, exon 6, affects transcript abundance. Strain means ± SEM. *R*^2^ and p values are strain averaged results; linear mixed model FDR <2.2 × 10^−16^. (F) RNA editing. *Cdc42bpb*, editing site chromosome 12, 111,309,987 bp in *Alu* element B1_Mus2, intron 21, FDR = 0.04; *Dock3*, editing site chromosome 9, 106,905,884 bp in *Alu* element B1_Mur1, intron 44/49, FDR = 0.04. See also Figures S3, S4, and S5.

The fixed effect of infusate was significant for a total of 5,111 transcripts, representing either induction or repression by cocaine (false discovery rate [FDR] < 0.05).^34^ Regulated genes included *Per2, Fam107a, Eif5*, and *Ankrd28* (Figures 3A and S4A). *Per2* is a core circadian rhythm gene known to be regulated by cocaine that, in turn, alters the effects of cocaine on circadian phase shifts.^35^ Gene Ontology (GO) analysis using the biological process term showed 465 significantly enriched functional categories in the transcripts regulated by infusate (FDR < 0.05) (Figure S4I). Metabolic process was prominent, including nitrogen compound and organic substance metabolic process. Genes related to cocaine and addiction were featured in these processes, including *Bche* and *Comt*, which are involved in cocaine metabolism, *Oprm1*, the μ opioid receptor, and *Cnr1*, a metabotropic cannabinoid receptor.^36^

Confirming our results, there was significant overlap between the regulated transcripts found in our investigation and a recent study that used RNA-seq to examine the NAc of C57BL/6J mice that underwent cocaine IVSA (odds ratio = 1.7, p = 6.2 × 10^−5^, Fisher’s exact test).^37^ We found significant regulation due to brain region or sex (supplemental information, Figures S4B and S4D). A total of 48 transcripts were identified with significant sex × infusate interactions (FDR < 0.05), including *Bc1, Crebzf, Taok1*, and *Psmc3* (Figures 3B and S4C). *Bc1* is a non-coding gene whose RNA is transported to dendrites to regulate translation.^38^

To identify factors that regulate splicing or RNA editing, we again used the linear mixed model implemented in lme4qtl. We added transcript abundance to the fixed effects of brain region, sex, infusate, sex × infusate interaction, and batch. A total of 31 exons showed significant differential splicing as a result of infusate (cocaine vs. saline; FDR < 0.05) (Figures 3C, 3D, and S4E).^39^ Examples included *Rbm39* and *Luc7I*, which themselves both regulate splicing.^40,41^ Reminiscent of *Per2, Rbm39* also shows a circadian rhythm-based splicing regulation.^42^ GO analysis of spliceforms regulated by cocaine revealed significant enrichment in terms related to RNA splicing (Figure S4J). A total of 860 differential splicing events were significantly associated with the fixed effect of transcript abundance due to exon selection affecting RNA stability (FDR < 0.05) (Figures 3E and S5A-S5C).^43^

Only two genes showed significant changes in RNA editing levels as a result of cocaine, *Cdc42bpb* and *Dock3* (FDR < 0.05) (Figures 3F and S4G). Both editing sites are in intronic *Alu* elements. GO analysis of genes in which RNA editing was nominally regulated by cocaine or cocaine × sex interactions (p < 0.05) showed significant enrichment of a number of categories including organelle organization and metabolic processes (Figure S4K).

Spliceforms and RNA editing events significantly regulated by brain region were also identified (FDR < 0.05) (supplemental information, Figures S4F, S4H, S4J, and S4K). A total of 13 RNA editing sites significantly regulated by brain region resulted in non-synonymous coding region changes, including *Cadps*, *Tmem63b, Unc80*, and *Cyfip2* (Figure S4H).

### Expression QTLs

**Figure 4.**
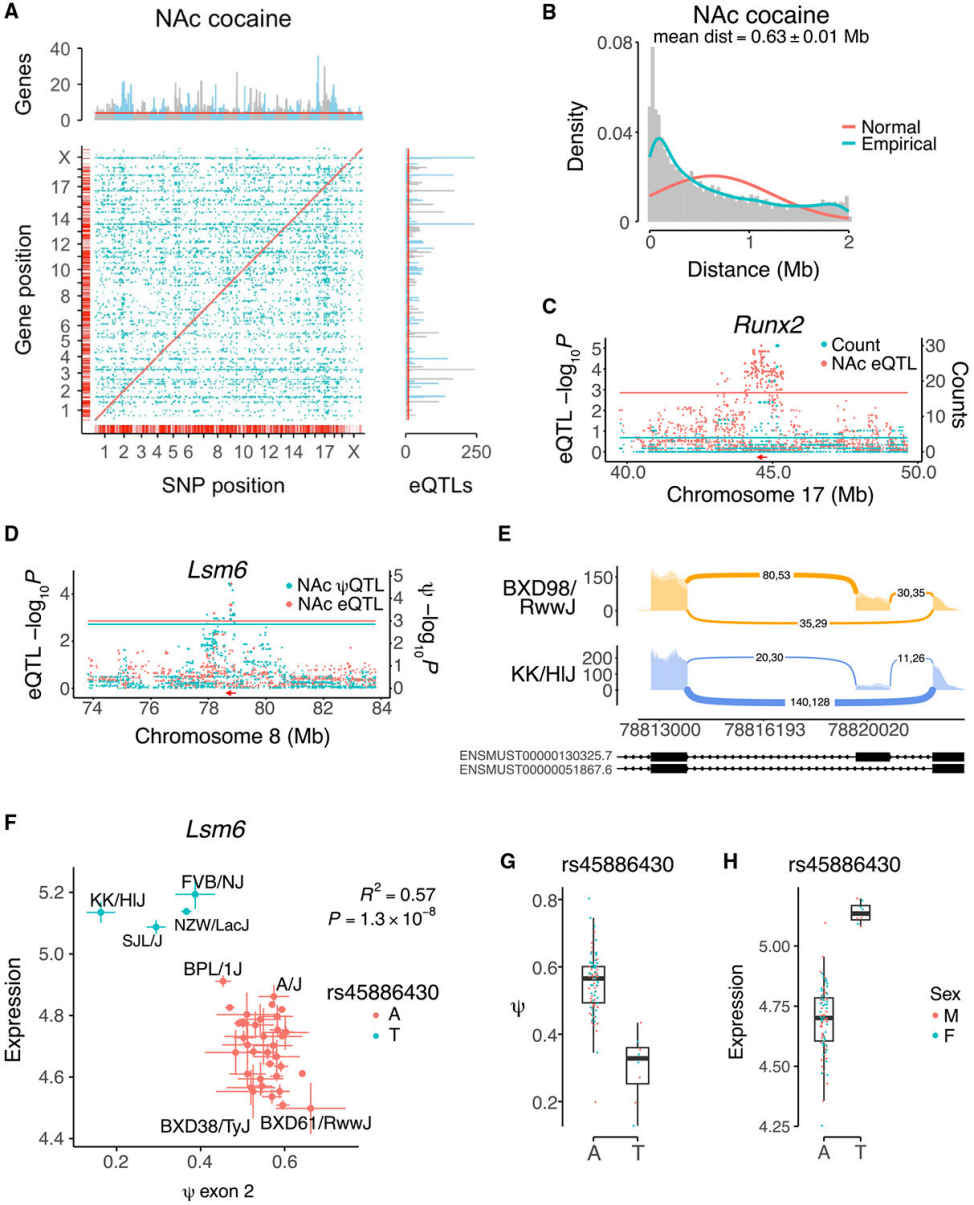
Genetic regulation of gene expression in NAc from cocaine-treated mice (A) *Cis* (red) and *trans* (blue) eQTLs. Marginal graphs show SNPs regulating many genes (horizontal graph, eQTL hotspots) and genes regulated by many eQTLs (vertical graph). Red lines, FDR < 0.05 (Poisson). (B) Distance between *cis* eQTLs and the corresponding genes. (C) Co-aligned eQTL hotspot and *Runx2 cis* eQTL. Red arrow, location of *Runx2*. Blue horizontal line, eQTL hotspot significance threshold, FDR < 0.05. Red horizontal line, *cis* eQTL significance threshold. (D) Coincident *cis* ψQTL for exon 2 and eQTL for *Lsm6*. Peak marker rs45886430 for both QTLs. Blue and red horizontal lines, respective significance thresholds. (E) Sashimi plot for exon 2 of *Lsm6* in BXD98/RwwJ and KK/HIJ, with A or T allele of rs45886430, respectively. (F) Allele A of peak marker rs45886430 is associated with higher ψ for exon 2 of *Lsm6* and lower expression. Normalized ψ and expression. Means ± SEM for each strain. (G) Allele effect of rs45886430 on ψ of *Lsm6* exon 2. Individual samples are shown. (H) Allele effect of rs45886430 on *Lsm6* expression. See also Figures S5 and S6.

*Cis* and *trans* QTLs were identified for transcript abundance (eQTLs), splicing (sQTLs or ψQTLs) and RNA editing (edit QTLs or φQTLs) using FaST-LMM (Figures 4A and S6A-S6C). The number of *cis* eQTLs averaged over the two brain regions and infusates was 4,844 ± 159 (Figures 4A and S6A). The distance between the *cis* eQTLs and their corresponding genes was 0.63Mb ± 0.005 Mb, averaged across brain regions and infusates, consistent with the known linkage disequilibrium structure of the HMDP (Figures 4B and S6D). Since enhancers >1 Mb from the regulated gene have been identified, with some as far away as 10 Mb,^44-49^ we chose to define *cis* eQTLs as those eQTLs residing closer than 2 Mb to the target gene. This cutoff is consistent with previous studies using the HMDP.^24,50,51^

We identified hotspots for transcript abundance, in which a locus regulates many genes.^24,51^ A total of nine hotspots regulating ≥20 genes were present in NAc cocaine samples (FDR < 2.2 × 10^−16^) and 10 hotspots in mFC cocaine samples (FDR < 2.2 × 10^−16^). We sought candidate genes for hotspots by looking for co-aligned *cis* eQTLs. One NAc cocaine hotspot was coincident with a *cis* eQTL for the transcription factor *Runx2* (Figure 4C).

### Splicing QTLs

A total of 1,426 ± 20 *cis* splicing QTLs (ψQTLs) were detected, averaged over the two brain regions and infusates (Figure S6B). Spliceforms regulated by genetic variants can affect transcript abundance as a result of changes in mRNA stability.^52,53^ To evaluate the prevalence of this phenomenon, we examined whether there was a statistically significant enrichment in coincident *cis* eQTLs and ψQTLs. There were 360 coincident *cis* eQTLs and ψQTLs in NAc from cocaine-treated mice (odds ratio [OR] = 2.8, p < 2.2 × 10^−16^, Fisher’s exact test), while cocaine-exposed mFC had 419 (OR = 2.6, p < 2.2 × 10^−16^). A coincident *cis* eQTL and ψQTL in cocaine-exposed NAc for *Lsm6*, a gene involved in pre-mRNA splicing,^54^ is shown in Figures 4D-4H.

### Editing QTLs

RNA editing results in sequence changes that can affect transcript stability and abundance as well as coding sequence.^55^ We identified 272 ± 11 *cis*-acting loci that affect RNA editing efficiency (φQTLs), averaged over the two brain regions and infusates (Figure S6C).^56^ Because RNA editing occurs at single nucleotides, confident quantitation of these events is more demanding than transcript or spliceform abundance. The ascertainment rate for all editing events was 37% ± 0.4% of RNA-seq samples, averaged across infusates and brain regions. The decreased power resulting from the <100% detection rate means that the φQTLs should be treated with some caution.

To evaluate how often genetically driven variations in RNA editing can affect transcript abundance, we tested for statistically significant increases in coincident *cis* eQTLs and φQTLs. There were 51 coincident *cis* eQTLs and φQTLs in NAc from cocaine-treated mice (OR = 2.0, p = 4.2 × 10^−5^, Fisher’s exact test), while cocaine-exposed mFC had 46 (OR = 1.6, p = 5.3 × 10^−3^). If *cis* φQTLs regulate editing events that in turn alter transcript stability and give rise to *cis* eQTLs, coincident *cis* φQTLs and *cis* eQTLs should be enriched in editing sites that appear in the final transcript rather than intronic or intergenic regions. This prediction was correct. We found significant enrichment of editing sites affecting 5′ untranslated, 3’ untranslated, and exonic coding regions in the coincident *cis* φQTLs and eQTLs in both cocaine-exposed NAc (odds ratio = 1.9, p = 9.2 × 10^−3^, Fisher’s exact test) and cocaine-exposed mFC (odds ratio = 2.9, p = 2.4 × 10^−5^, Fisher’s exact test).

A *cis* φQTL that regulates editing of a site in a B1_Mm *Alu* element in the 3′ untranslated region of *Samd8* (chromosome 14, 21,797,711 bp) and that aligns with a *cis* eQTL in NAc from cocaine-exposed mice is shown in Figures S5D-S5G.

### Integrating RNA-seq and behavioral loci

*Cis* eQTLs were used to narrow down the candidate genes for the longitudinal behavioral loci. A total of 17.4 ± 2.9 cocaine-exposed NAc *cis* eQTLs and 15.8 ± 2.3 cocaine-exposed mFC *cis* eQTLs lay within 2 Mb of each behavioral locus. Plausible candidate genes that aligned with either cocaine-exposed NAc or mFC *cis* eQTLs were found in seven of the 15 unique IVSA loci (Table 1).

**Figure 5.**
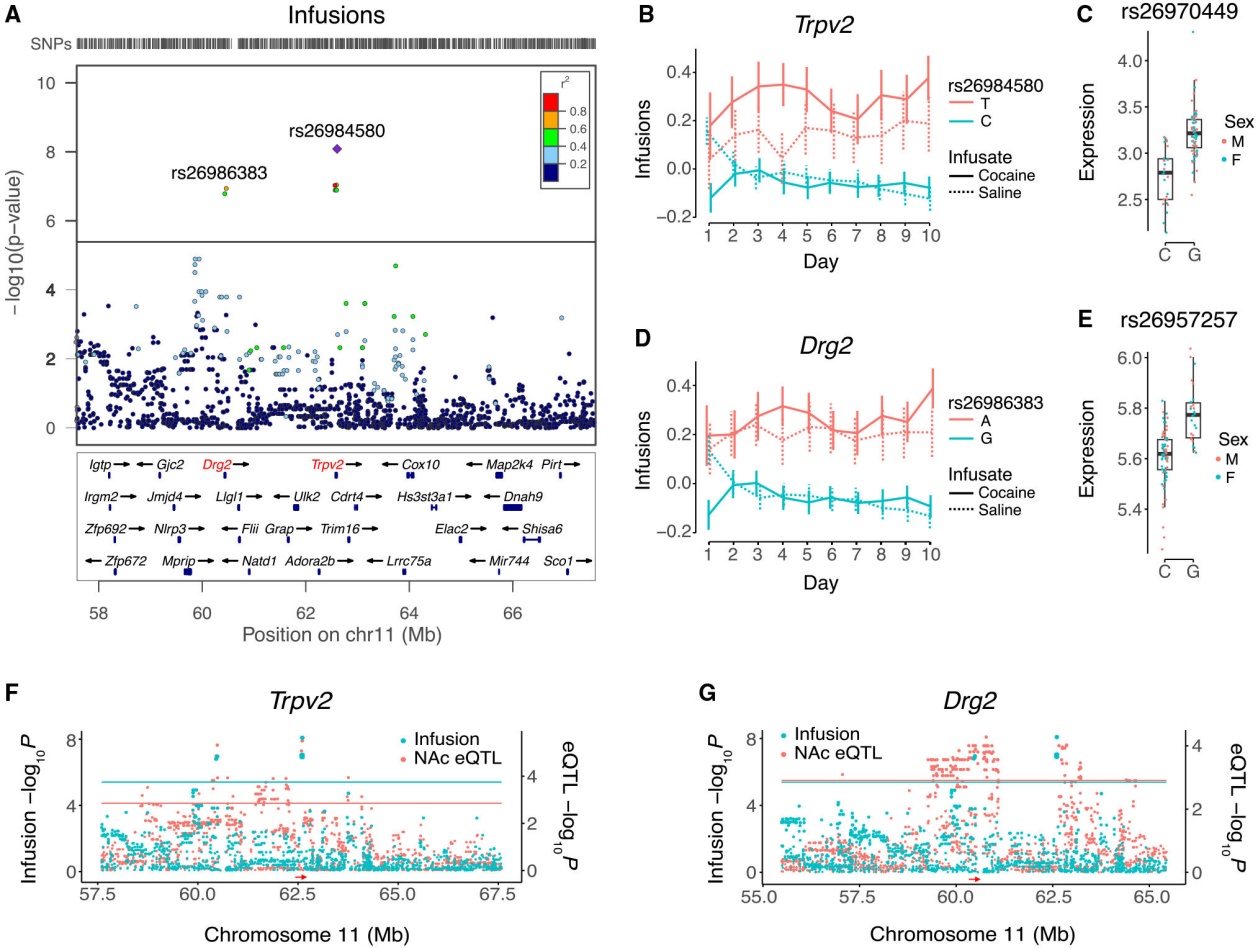
*Trpv2* and *Drg2* are close to infusion longitudinal QTLs and have *cis* eQTLs (A) LocusZoom plot for cocaine infusions, showing loci harboring *Drg2* and *Trpv2*. *R*^2^ values convey linkage disequilibrium. (B) Normalized infusion time course for peak SNP of *Trpv2* locus, rs26984580. Allele effect for saline not significant using longitudinal model. Means ± SEM. (C) *Trpv2* expression in cocaine-exposed NAc associated with peak *cis* eQTL SNP, rs26970449. SNPs rs26984580 and rs26970449 are in linkage disequilibrium (D’ = 1, *R*^2^ = 0.71, p < 2.2 × 10^−16^). (D) Infusion time course for peak SNP of *Drg2* locus, rs26986383. Saline allele effect is not significant. (E) Expression of *Drg2* for peak *cis* eQTL SNP, rs26957257, in cocaine-exposed NAc. SNPs rs26986383 and rs26957257 are in linkage disequilibrium (D’ = 1, *R*^2^ = 0.07, p = 1.8 × 10^−9^). (F) Coincident loci for infusions and *Trpv2 cis* eQTL in cocaine-exposed NAc. (G) Coincident loci for infusions and *Drg2 cis* eQTL in cocaine-exposed NAc. See also Figure S3.

The two most significant loci for cocaine infusions mapped to chromosome 11 at 60,484,778 bp and 62,605,569 bp (Figures 2A, 5, and S3A). Candidate genes for the two loci were *Drg2* and *Trpv2*, respectively, each of which were supported by significant *cis* eQTLs in both NAc and mFC (Figure 5).^57^ Higher expression of both *Drg2* and *Trpv2* was associated with lower cocaine infusions. The effect sizes of the two loci on infusions (0.33 ± 0.06, *Drg2*; 0.37 ± 0.07, *Trpv2*) were comparable to the difference between cocaine and saline (0.23 ± 0.02) (Figure S1B). There was, however, significant linkage disequilibrium between the *Drg2* and *Trpv2* loci (*D*’ = 0.91, *R*^2^ = 0.67, p < 2.2 × 10^−16^), making it hard to disentangle their relative contributions. Other genes supported by co-aligned *cis* eQTLs in the cocaine IVSA loci were *A630001G21Rik, Plch1*, *Asic2, Rnf17*, and *Cldn20* (Table 1).

### Transcriptome-wide association studies

We used TWASs to further evaluate genes for longitudinal cocaine IVSA. The TWAS approach nominates a gene for a trait if the gene possesses a *cis* eQTL and also shows significant correlation of its expression with the trait. Because TWAS employs genes rather than markers, there is decreased multiple hypothesis correction and thus increased statistical power. We used FUSION and FOCUS software to perform TWASs. Compared to FUSION, FOCUS provides fine mapping by controlling for both linkage disequilibrium and pleiotropy.^58,59^

**Figure 6.**
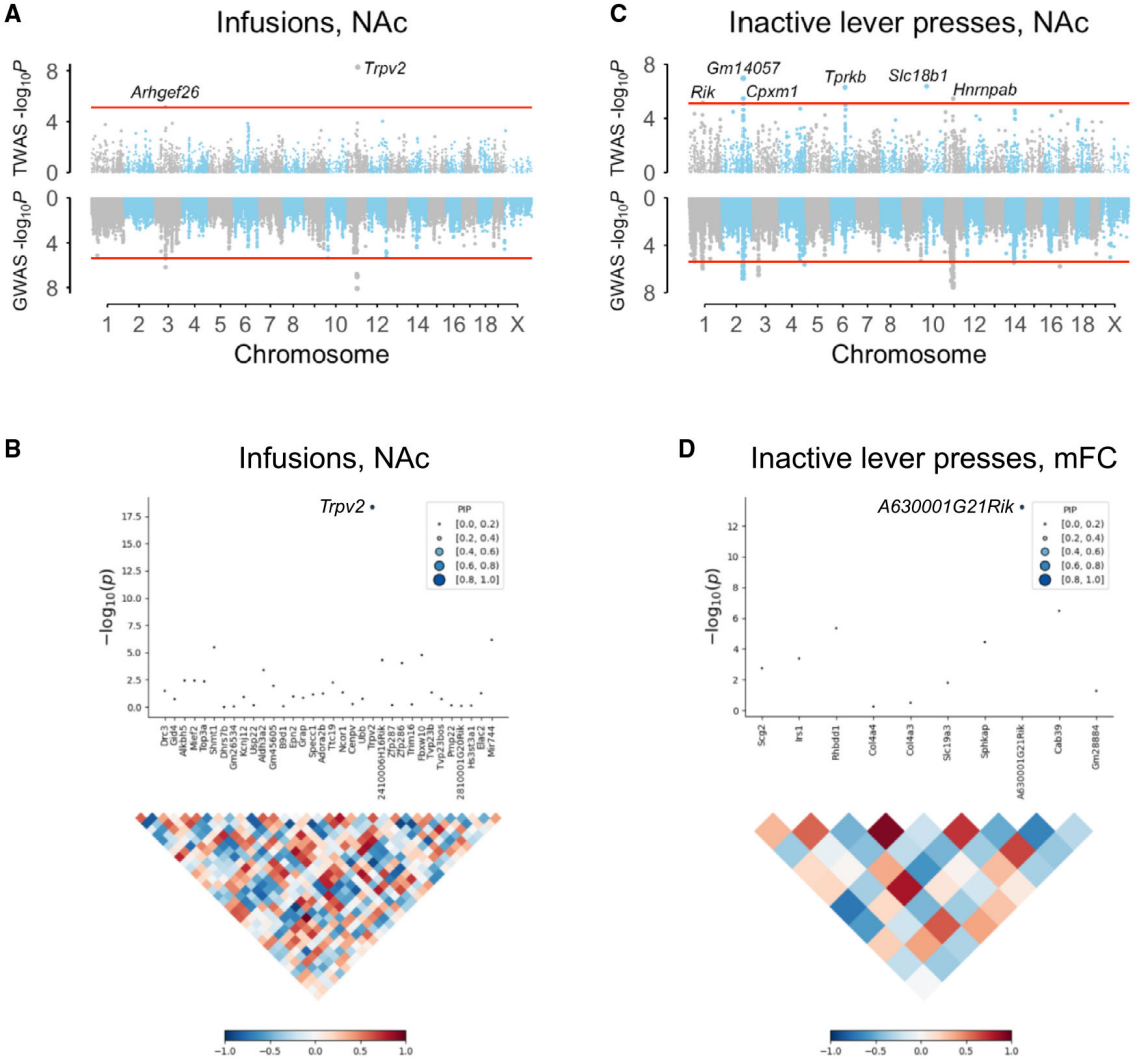
TWASs of cocaine IVSA (A) FUSION TWAS of infusions. *Trpv2* in NAc significant. (B) FOCUS of *Trpv2* for infusions in NAc (pip = 1.00). Linkage disequilibrium map is shown underneath. (C) FUSION TWAS of inactive lever presses in NAc. *Rik, A630001G21Rik*. (D) FOCUS of *A630001G21Rik* for inactive lever presses in mFC (pip = 0.91). All TWASs employ RNA-seq data from cocaine-exposed mice. See also Figure S7 and Table S2.

Consistent with its increased statistical power, TWAS identified 20 significant genes for the four behavioral endpoints of cocaine IVSA. Of these genes, three were present in the longitudinal GWASs (*A630001G21 Rik, Gna12*, and *Trpv2*) and 17 were new (Tables 1 and S2 and Figures 6 and S7A-S7E). Of the 20 TWAS significant genes, 12 were significant using FUSION (*Slco5a1, Cpxm1, Gm14057, Arhgef26, Tprkb, Slc18b1, Mgat4b, Hnrnpab, Gdi2, Trat1, Dubr*, and *Gm10232*), five were significant using FOCUS (*Slc4a11, Gna12, 9930111J21Rik2, Gm12216*, and *Mief2*), and three (*A630001G21Rik, G3bp1*, and *Trpv2*) were common to both.

In some cases, TWAS suggested different genes than those nominated on the basis of proximity and biological plausibility (Table 1). For example, *G3bp1* was significant for cocaine inactive lever presses using both FUSION (p = 1.83 × 10^−6^) and FOCUS (posterior inclusion probability, pip = 0.91) on RNA-seq data from cocaine-exposed mFC. However, *G3bp1* was 626,581 bp from the nearest behavioral QTL. In contrast, the nominated candidate gene for this QTL, *Hint1*, was 7,761 bp from the locus. Although *Hint1* had no significant *cis* eQTLs, we gave this gene precedence because of its proximity to the behavioral QTL and its known role in addiction.^60,61^ Further work is required to distinguish which of the two genes (or both) is relevant to cocaine IVSA. A similar situation exists for *Pdyn* and *Cpxm1*, and *Drg2* and *Mief2*. Regardless, the TWAS genes for cocaine IVSA are useful entry points for new studies of drug addiction.

Among the three genes supported by TWAS in the 15 non-redundant cocaine IVSA loci, FUSION and FOCUS provided strong evidence for *Trpv2* (Tables 1 and S2). The TWAS results for *Trpv2* were significant for both cocaine infusions and active lever presses using cocaine-exposed NAc data but not mFC. However, the neighboring candidate gene *Drg2* was not supported by TWASs, despite significant *cis* eQTLs in cocaine-exposed NAc and mFC. *Drg2* and *Trpv2* show significant linkage disequilibrium, but the linkage is less than perfect, leaving room for discordant TWAS results. Further, environment may affect *Drg2* expression differently than *Trpv2*, weakening any correlation of *Drg2* expression levels with cocaine self-administration.

Additional strain transcriptomes may provide enough power to support a role for *Drg2* in cocaine IVSA using TWASs. Alternatively, *Drg2* may exert its effects through amino acid variations rather than expression, although no such variants are currently known among the 37 sequenced inbred mouse strains.^62,63^ Genome sequencing of further strains in the HMDP may reveal *Drg2* protein-altering variants.

### eCAVIAR

To further dissect the contributions of *Trpv2* and *Drg2* to cocaine IVSA, we used eCAVIAR. This software evaluates the posterior probability that the same SNP is causal for both GWAS and expression QTLs, while accounting for the uncertainty introduced by linkage disequilibrium.^64^ A colocalization posterior probability (CLPP) > 0.01 supports sharing of causal GWAS and eQTL variants. eCAVIAR gives superior performance to conditional analysis, which relies on iterative selection of the most significantly associated SNPs but can lead to selection of no causal SNPs if the markers are in high linkage disequilibrium.

Consistent with the TWAS results, *Trpv2* had above-threshold CLPPs for infusions (NAc, rs26984580, CLPP = 0.13; mFC, rs26970449, CLPP = 0.03) and active lever presses (NAc, rs26984580, CLPP = 0.06; mFC, rs26970449, CLPP = 0.02), while *Drg2* did not. CLPPs for *Trpv2* were higher in cocaine-exposed NAc than mFC, suggesting NAc is the more relevant target tissue. This observation accords with the significant TWAS results for *Trpv2* in NAc but not mFC (Table 1). The SNP with the highest CLPP for *Trpv2* (rs26984580) was 18,069 bp telomeric to *Trpv2*. The SNP is located midway (180 bp) between two transcriptional regulatory elements separated by 362 bp in the 3′ untranslated region of the neighboring gene, *Lrrc75a*.

The fact that eCAVIAR, which employs a different statistical approach than TWAS, gives similar discordant results for *Trpv2* and *Drg2* is further support for *Trpv2* as the relevant candidate gene for cocaine self-administration.

## DISCUSSION

Longitudinal GWASs increased the power to detect QTLs affecting cocaine IVSA. The longitudinal GWASs identified 15 unique loci using the four IVSA endpoints, of which three loci were for cocaine infusions. GWASs using individual days identified no significant loci, while GWASs using binned 2-day intervals identified two significant loci and one suggestive locus for infusions.^25^ Further, the longitudinal GWAS QTLs had higher peak −log_10_p values than the GWASs using 2-day intervals.

The GWASs using cocaine infusion data binned over 2 days identified a suggestive QTL on chromosome 3 (36,771,265 bp) and a significant QTL on chromosome 14 (56,388,089 bp). Both QTLs are within credible linkage disequilibrium distances of loci identified using the longitudinal model. The suggestive 2-day QTL on chromosome 3 is 1,406,935 bp centromeric to a longitudinal QTL for inactive lever presses, which has *Spry1* as the candidate gene (Table 1). *Spry1* falls between the binned 2-day QTL (871,007 bp telomeric) and the longitudinal QTL (535,928 bp centromeric). The 2-day QTL on chromosome 14 is 8,077 bp telomeric to a longitudinal QTL for inactive lever presses, with *Rnf17* as the candidate gene. *Rnf17* is supported by a *cis* eQTL in mFC.

Coincident *cis* eQTLs and TWASs gave support to longitudinal GWAS candidates, while suggesting additional genes. Only three significant TWAS genes were common to FUSION and FOCUS. In contrast to FUSION, which treatsgenes independently, FOCUS allows for fine mapping of TWAS results by controlling for linkage disequilibrium, while also accounting for pleiotropic effects. Moreover, the performance of FOCUS is preserved in proxy tissues whose expression is correlated with the causative tissue.

The role of *Trpv2* in cocaine IVSA was supported by *cis* eQTLs in NAc and mFC, as well as TWASs and eCAVIAR. The TWAS and eCAVIAR analyses suggested NAc was a more relevant target tissue for *Trpv2* than mFC. *Trpv2* is a cation channel that is activated by cannabidiol and Δ9-tetrahydrocannabinol, possibly explaining the overlapping genetic risk factors for cocaine and cannabis use disorders.^6,65,66^ In fact, the six known Trp cation channels are sometimes referred to as ionotropic cannabinoid receptors. Cannabidiol shows promise as a treatment for cocaine use disorder, consistent with our observation that higher expression of *Trpv2* is associated with lower cocaine IVSA.^67^ *Trpv2* knockout mice have been created and show macrophage and mechanical nociception phenotypes.^68-70^ These mice would be an attractive reagent to test the role of *Trpv2* in cocaine IVSA.

Support for *Drg2* as a gene for cocaine IVSA was provided by *cis* eQTLs in NAc and mFC. Consistent with a potential role in cocaine addiction, *Drg2* knockout mice show decreased dopamine release in the striatum.^71^ However, *Drg2* was not supported by TWASs or eCAVIAR. A locus for inactive lever presses was close to *A630001G21Rik*, a likely nuclear body protein involved in transcription, and was supported by NAc and mFC *cis* eQTLs and also TWASs.^72^

Another locus for inactive lever presses was close to *Pdyn*. Although not supported by *cis* eQTLs or TWASs, *Pdyn* encodes prodynorphin, which is proteolytically processed to produce κ opioid receptor ligands and may cause aversion to cocaine.^73,74^ There is a body of literature showing upregulation of *Pdyn* following chronic cocaine administration.^75^ However, neither our study nor a recent RNA-seq study of the NAc in C57BL/6J mice self-administering cocaine^37^ found significant upregulation of *Pdyn*.

Candidate genes lacking additional supporting evidence in the current study are speculative. For example, the peak SNP for the locus on chromosome 12 regulating the number of active presses (rs33619289) is actually located in the middle of the immunoglobulin heavy chain complex, near variable region gene 3–48 (*Ighv1–43*). The nominated candidate gene, *Vipr2*, is located 1,156,141 bp telomeric to the SNP, and another gene, *Ptprn2*, is 1,925,431 bp telomeric.

*VIPR2* and *PTPRN2* were significant in a linkage analysis of comorbid cocaine dependence and major depressive episode in humans.^76^ Further, *Ptprn2* is significant in human GWASs for cognitive performance, risk taking, and smoking initiation.^77^ Copy number increases in *Vipr2* are associated with schizophrenia and alter dopaminergic neurotransmission in engineered mice.^78^ *Ptprn2* knockout mice show decreased brain dopamine, norepinephrine, and serotonin concentrations.^79^

In our study, *Vipr2* had no *cis* eQTLs, while *Ptprn2* had significant *cis* eQTLs in both cocaine-exposed NAc and mFC. We nominated *Vipr2* rather than *Ptprn2* largely because *Vipr2* is substantially closer to the peak behavior SNP. Although both genes are biologically plausible and within the credible distance for linkage disequilibrium, their candidacy should be treated with caution.

*Slc18b1* was significant for inactive lever presses using FUSION analysis of RNA-seq data from both cocaine-exposed NAc and mFC. *Slc18b1* is a serotonin transporter, consistent with the role of cocaine in inhibiting the reuptake of this neurotransmitter.^80^ Other TWAS significant genes included *Cpxm1*, also significant in human GWASs for cognitive and executive function; *Arhgef26* for body mass index, sulcal depth, and smoking initiation; *G3bp1* for cortical thickness; and *Dubr* for worry.^77^

We used geneMANIA, a repository of gene interactions, to chart links between the 32 candidate genes uncovered by the longitudinal behavioral GWASs and TWASs (Figure S7F).^81^ There were 90 interactions involving the cocaine IVSA genes, featuring 25 candidate genes and 20 additional genes. For example, *Rnf17, Pdyn*, and *Hnrnpab*, candidate genes for inactive lever presses (Figure 6 and Table 1), showed genetic interactions.^82^

### Limitations of the study

Our study lacks experimental confirmation of the candidate genes. However, at least eight of the candidate genes have been evaluated in knockout mice for traits other than addiction (*Vipr2, Pdyn, Asic2, Hint1, Drg2, Spry1, Rnf17, Trpv2*).^70^,^71^,^83-88^ Expanding the phenotyping of these mice to include cocaine IVSA would be of value.

The single-day GWAS and heritability data suggest that saline longitudinal loci will show illuminating differences with cocaine. Consistent with these observations, only one out of 17 peak significant SNPs for longitudinal cocaine IVSA were also significant for longitudinal saline IVSA. However, a fuller picture awaits the completion of saline longitudinal GWASs, which are ongoing. Recently, a large number of new BXD RI strains have been constructed and genotyped, bringing the total to 140 strains.^89^ Adding these mice to the study of cocaine IVSA will increase power and reveal further loci.

Dose-dependent gene regulation by cocaine or saline could be evaluated in the lme4qtl linear mixed model using self-administration measures as continuous rather than categorical variables. Adult NAc and mFC may not be the optimal tissues to identify *cis* eQTLs linked to cocaine IVSA. Analysis of various developmental time points in other brain regions, such as amygdala or ventral tegmental area, may be more relevant. TWAS relies on filtering by *cis* eQTLs. In contrast, reference trait analysis employs both environmental and genetic variation to relate transcript levels to phenotypes and can outperform TWASs.^90^ For genes lacking *cis* eQTLs, TWASs could be implemented using *cis* ψQTLs and *cis* φQTLs.

Pathways for cocaine addiction can be identified from the RNA-seq data using tools such as KEGG and WikiPathways.^91,92^ Phenome-wide association studies (PheWASs) interrogate individual genetic variants for their effects on multiple phenotypes.^93^ Combining the cocaine IVSA data with phenotypes already ascertained in the HMDP^23^ will be a rich resource to illuminate addiction mechanisms using PheWASs.

## STAR★METHODS

### RESOURCE AVAILABILITY

#### Lead contact

Further information and requests for resources and reagents should be directed to and will be fulfilled by the lead contact, Desmond J. Smith (DSmith@mednet.ucla.edu).

#### Materials availability

This study did not generate new unique reagents.

#### Data and code availability

- This paper analyzes existing, publicly available data. The accession numbers for the datasets are listed in the key resources table.
- All original code has been deposited at figshare: https://doi.org/10.6084/m9.figshare.21539487 and is publicly available as of the date of publication. DOIs are listed in the key resources table.
- Any additional information required to reanalyze the data reported in this paper is available from the lead contact upon request.

### EXPERIMENTAL MODEL AND SUBJECT DETAILS

#### Mice

A total of 479 and 477 mice from 84 strains of the HMDP were used for cocaine and saline IVSA respectively, as described earlier.^25^ Animals for IVSA were acquired from the Jackson Laboratory (Bar Harbor ME) with an indwelling jugular catheter. A total of 32 inbred and 52 recombinant inbred strains were evaluated. Target numbers were 3 males and 3 females for each strain for each of the two infusates. The actual number per strain was 5.7 ± 0.1 s.e.m. for both cocaine and saline, exceeding the 5 animals calculated to provide 80% power to identify a QTL with an effect size of 10% in the 100 strains of the HMDP.^104^ There were close to equal numbers of males and females within each strain and infusate (50.4 ± 0.7% males). The age of the mice was 11.3 ± 0.07 weeks. Mouse experiments were approved by the Binghamton University Institutional Animal Care and Use Committee and conformed to all relevant regulatory standards.

### METHOD DETAILS

#### Cocaine intravenous-self administration

Mice were subjected to either cocaine or saline IVSA over 10 consecutive daily sessions. Animals were confronted with two levers in the testing chambers, one of which gave an infusion of cocaine or saline (active lever), the other of which did not (inactive lever). A time-out period of 20 s occurred after an infusion, during which active lever presses were recorded but no infusion was given. Testing continued until 65 infusions were administered or 2 h elapsed, whichever came first. The amount of free base cocaine administered per infusion was 0.5 mg kg^−1^ of body weight. Consequently, the behavioral endpoints represent normalized cocaine doses. The concentration of sterile saline was 0.84 mg mL^−1^. The infusion volume was 0.67 mL kg^−1^ infusion^−1^ for both cocaine and saline.

Four endpoints were analyzed; number of infusions, number of active lever presses, percentage of active lever presses and number of inactive lever presses. The first three endpoints evaluate the propensity of the mice for cocaine self-administration. Percent active lever presses control for locomotor activity by normalizing active lever presses to total lever presses. In contrast, inactive lever presses may measure either the aversive properties of the infusate or locomotor activity, whether intrinsic or modified by infusate.

#### RNA-seq

NAc (core and shell) and mFC were harvested from all mice 24 h after their final test session.^25^ RNA-seq was performed on the first 41 strains exposed to either cocaine or saline and consisted of 28 inbred (A/J, AKR/J, LP/J, NOD/ShiLtJ, 129X1/SvJ, BALB/cByJ, BALB/cJ, BPL/1J, C3H/HeJ, C3HeB/FeJ, C57BL/10J, C57BL/6J, C57BLKS/J, C57BR/cdJ, C58/J, CBA/J, DBA/1J, DBA/2J, FVB/NJ, KK/HlJ, MA/MyJ, MRL/MpJ, NZB/BlNJ, NZO/HlLtJ, NZW/LacJ, PL/J, SJL/J and SM/J) and 13 recombinant inbred strains (BXD31/TyJ, BXD32/TyJ, BXD38/TyJ, BXD40/TyJ, BXD42/TyJ, BXD48a/RwwJ, BXD61/RwwJ, BXD62/RwwJ, BXD65/RwwJ, BXD73a/RwwJ, BXD77/RwwJ, BXD84/RwwJ and BXD98/RwwJ).

RNA-seq used individual samples for four strains (A/J, AKR/J, LP/J, NOD/ShiLtJ), resulting in 6 samples composed of 3 males and 3 females for each infusate and brain region. For the remaining strains, we pooled samples of the same sex, yielding a total of 2 samples (male or female) for each infusate and brain region. Samples were pooled to save library construction costs, while preserving information on sex, infusate and brain region. The total number of RNA-seq samples was 392. Sequencing used 75 bp paired-ends for cocaine with 72.6 ± 1.1 million (M) reads per strain and brain region and 73.0 ± 1.1 M for saline.

Reads were mapped as described^25,51^ to mouse genome sequence build GRCm38.p6 downloaded from Ensembl^96^ using STAR aligner.^103^ Alignments employed the default mismatch value of 10, permitting one multiple mapping for each read. The transcriptome used was Gencode M25 (GRCm38.p6).^98^ Expression levels at the gene level were obtained from each sample by using htseq-count to evaluate the aligned and sorted BAM files produced from STAR.^101^

### QUANTIFICATION AND STATISTICAL ANALYSIS

#### Behavioral covariates

The effects of the covariates on the normalized behavioral phenotypes were evaluated using a linear mixed model implemented in lme4 with day of assay, sex, chamber number, active lever and age as fixed effects. Day of assay and age were treated as continuous variables. The model assessed population structure using strain as a random effect.^26,102^

#### Heritability

Broad sense heritability (H^2^) was calculated using the same model as the behavioral covariates, but with day of assay omitted to evaluate H^2^ on individual days. Additive heritability (h^2^) was calculated using the heritability package.^99^ All heritability analyses used normalized data.

#### Genome-wide association studies of IVSA

Loci for cocaine and saline IVSA on individual days were mapped using a linear mixed model implemented in FaST-LMM to correct for population structure via a kinship matrix.^25,27^ Covariates included sex, active lever (left or right), testing chamber, cohort and age. Behavioral data used individual mice and were normalized within individual days using the rank-based inverse normal transformation (Blom’s method).^105^ Tied values were replaced by their mean. Genome-wide significance thresholds were obtained from permutation, p < 4.1 × 10^−6^, as described.^51,104^ The corresponding family-wide error rate was 5%. Single nucleotide polymorphism (SNP) genotypes were obtained from the mouse diversity array.^94^ After removing SNPs with minor allele frequency <5% or missing genotype frequency >10%, 340,097 SNPs remained for mapping. Coordinates are from mouse genome build GRCm38/mm10.^95^

To increase statistical power, we used a linear mixed model implemented in GMMAT to evaluate the fixed effects of SNPs on the normalized IVSA phenotypes treated as longitudinal traits.^28^ The approach effectively used individual mice as repeated measures, preserving valuable degrees of freedom and giving increased power compared to strain means. The model employed fixed and random effects of testing day as a continuous variable and also corrected for population structure using random effect of genotype via a kinship matrix. Normalization and covariates were the same as for FaST-LMM.

Incorporating an infusate × SNP interaction term into the model would allow efficient identification of loci that differentially affect cocaine and saline IVSA. However, we were unable to find available software that would accomplish this goal while incorporating other necessary model features. Further, a sample size increase of ~16-fold is required to detect an interaction with the same power as a main effect, and an underpowered term can lead to unacceptable false positive and false negative rates.^33^

Significance testing used the Wald test, because of its increased power compared to the score test.^106^ The GMMAT model used the same threshold p < 4.1 × 10^−6^ as for FaST-LMM. One genome scan took ~4 weeks on a computer cluster continuously running ~100 nodes in parallel, each using 16 Gb of memory consisting of 4 cores of 4 Gb.

#### GWAS of transcript abundance

Gene transcripts with ≥6 reads and transcripts per million (TPM) >0.1 in at least 20% of samples for each infusate (cocaine or saline) and brain region (NAc or mFC) were selected for GWAS.^51,107^ A total of 21,118 ± 41 transcripts remained for analysis, averaged over the two brain regions and infusates. Conditional quantile normalization was used to normalize the data.^108^ We mapped expression quantitative trait loci (eQTLs) separately for cocaine and saline using FaST-LMM with covariates of sex and sequencing batch.

*Cis* eQTLs were defined as residing within 2 Mb of the regulated gene. Genome-wide significance thresholds of p < 1.4 × 10^−3^ for *cis* eQTLs and 6 × 10^−6^ for *trans* were derived from permutation, corresponding to a family-wise error rate of 5%.^51^ Pairs of QTLs, whether behavioral or molecular, were defined as coincident if they were located within 2 Mb of each other.

#### GWAS of splicing

Read mapping for spliceforms was performed as for transcripts using STAR and htseq-count, followed by calculation of percentage spliced in (psi, or ψ) at the exon level.^109^ Although many packages are available to quantitate splicing,^110^ we chose STAR and htseq-count because of their wide popularity. Exons were selected with ≥5 reads in all samples for each infusate and brain region. For each transcript, the exon with the highest standard deviation of percentage spliced in (psi, or ψ) between individuals was chosen.^111^ Of these, only exons with non-zero standard deviation that were also included in the transcript abundance analysis were retained. A total of 9,436 ± 59 exons remained for analysis, averaged over the two brain regions and infusates. Values of ψ were quantile normalized and QTLs for alternate splicing (ψQTLs) mapped using FaST-LMM.

#### GWAS of RNA editing

We quantified RNA editing sites by aligning RNA-seq reads using hisat2 v.2.0.4 with default parameters.^100^ Unmapped reads were realigned using a pipeline to resolve mapping of hyper-edited reads.^112,113^ RNA editing sites were then obtained from the REDIportal database and downstream processing steps performed as described.^97,114-116^

Editing sites were retained for further analysis if ≥ 10% of samples for each infusate and brain region had data. A total of 5,266 ± 181 editing sites were analyzed, averaged over the two brain regions and infusates. We quantile normalized editing ratios (φ) and used FaST-LMM to map QTLs regulating RNA editing (φQTLs). All editing sites were A to I.

#### Regulation of transcripts, spliceforms and editing sites

To identify expression changes independent of genetic background, we used a linear mixed model implemented in lme4qtl.^32^ Fixed effects were brain region, sex, infusate, sex × infusate interaction and RNA-seq batch. For splicing and editing, transcript expression level was added as an additional fixed effect. Random effects used a kinship matrix to account for population structure and correct for regulatory effects due solely to genetic background.

#### Transcriptome-wide association studies

Transcriptome-wide association studies (TWAS) were performed using FUSION and FOCUS software.^58,59^ Significance thresholds for FUSION used p < 0.05, Bonferroni corrected for the number of genes tested. FOCUS genes were considered significant if the posterior inclusion probability (pip) > 0.8.

#### eCAVIAR

To find SNPs that co-regulated *cis* eQTLs and behavioral loci with the highest colocalization posterior probability (CLPP), we used eCAVIAR to evaluate markers within 200 SNPs of the *cis* eQTL.^64^ A CLPP >0.01 is considered the support threshold for a co-regulating SNP.

## Supplementary Material

1

2

## Figures and Tables

**Table 1.** Loci for cocaine self-administration

Chr	SNP	Position (bp)	*P*	Assay	Gene	Evidence^a^			
						Distance (bp)^b^	FUS^c^	FOC^d^	*Cis* eQTL^e^
1	rs33037178	85,670,029	1.1E-06	Inact	*Rik* ^f^	77,834	NAc	mFC	both^g^
2	rs28034317	79,739,802	2.6E-06	% Act Press	*Ppp1r1c*	23,336	N/A	N/A	N/A
2	rs27257529	129,620,509	1.6E-07	Inact	*Pdyn*	72,695	N/A	N/A	N/A
3	rs30059671	38,178,200	3.2E-07	Inact	*Spry1*	−535,928	N/A	N/A	N/A
3	rs50587939	63,815,060	6.6E-07	Infus	*Plch1*	−17,207	N/A	N/A	mFC
4	rs32355822	153,414,160	2.3E-06	Inact	*Ajap1*	13,856	N/A	N/A	N/A
5	rs6393330	138,877,875	3.0E-06	% Act Press	*Gna12*	1,916,545	N/A	NAc	N/A
11	rs26988786	54,860,681	2.9E-08	Inact	*Hint1*	7,761	N/A	N/A	N/A
11	rs26942304	60,457,090	2.8E-06	Act Press	*Drg2*	4,582	N/A	N/A	both
11	rs26986383	60,484,778	1.1E-07	Infus	*Drg2*	−23,106	N/A	N/A	both
11	rs26984580	62,605,569	3.2E-06	Act Press	*Trpv2*	−18,069	NAc	NAc	both
11	rs26984580	62,605,569	8.3E-09	Infus	*Trpv2*	−18,069	NAc	NAc	both
11	rs28241639	81,647,142	3.4E-06	% Act Press	*Asic2*	−222,829	N/A	N/A	mFC
12	rs33619289	114,955,853	2.4E-07	Act Press	*Vipr2*	1,156,141	N/A	N/A	N/A
13	rs29602391	21,073,361	3.2E-06	% Act Press	*Trim27*	113,723	N/A	N/A	N/A
14	rs48220977	56,380,012	3.3E-06	Inact	*Rnf17*	83,794	N/A	N/A	mFC
17	rs33126598	4,133,471	1.7E-06	Inact	*Cldn20*	−600,587	N/A	N/A	NAc

Infus, number of infusions; Act Press, active lever presses; % Act Press, percent active lever presses; Inact, inactive lever presses.

a In the absence of other supporting evidence, candidate genes were nominated based on a combination of proximity and biological function.

b Negative distance, gene centromeric to SNP; positive, telomeric.

c FUSION in cocaine-exposed mice.

d FOCUS in cocaine-exposed mice.

e *Cis* eQTLs in cocaine-exposed mice.

f Rik, A630001G21Rik.

g Both NAc and mFC.

**Table:** KEY RESOURCES TABLE

REAGENT or RESOURCE	SOURCE	IDENTIFIER
Deposited data		
Data and code	This paper	https://doi.org/10.6084/m9.figshare.21539487
Mouse diversity array genotypes	Rau et al., 2015^94^	https://phenome.jax.org/projects/CGD-MDA1
Mouse genome build GRCm38/mm10 for genotypes	Lee et al., 2022^95^	https://genome.ucsc.edu/
Mouse genome sequence build GRCm38.p6 for transcriptome	Howe et al., 2021^96^	https://nov2020.archive.ensembl.org/Mus_musculus/Info/Index
REDIportal	Picardi et al., 2017^97^	http://srv00.recas.ba.infn.it/atlas/
RNA-seq data	Bagley et al., 2022^25^	https://www.ncbi.nlm.nih.gov/bioproject/; accession number PRJNA755328
Transcriptome, Gencode M25	Frankish et al., 2021^98^	https://www.gencodegenes.org/mouse/release_M25.html
Experimental models: Organisms/strains		
Mouse: 129S1/SvlmJ	The Jackson Laboratory	Cat# 002448; RRID: IMSR_JAX:002448
Mouse: 129X1/SvJ	The Jackson Laboratory	Cat# 000691; RRID: IMSR_JAX:000691
Mouse: A/J	The Jackson Laboratory	Cat# 000649; RRID: IMSR_JAX:000649
Mouse: AKR/J	The Jackson Laboratory	Cat# 000648; RRID: IMSR_JAX:000648
Mouse: BALB/cByJ	The Jackson Laboratory	Cat# 001026; RRID: IMSR_JAX:001026
Mouse: BALB/cJ	The Jackson Laboratory	Cat# 000651; RRID: IMSR_JAX:000651
Mouse: BPL/1J	The Jackson Laboratory	Cat# 003006; RRID: IMSR_JAX:003006
Mouse: BTBRT+Itpr3tf/J	The Jackson Laboratory	Cat# 002282; RRID: IMSR_JAX:002282
Mouse: C3H/HeJ	The Jackson Laboratory	Cat# 000659; RRID: IMSR_JAX:000659
Mouse: C3HeB/FeJ	The Jackson Laboratory	Cat# 000658; RRID: IMSR_JAX:000658
Mouse: C57BL/10J	The Jackson Laboratory	Cat# 000665; RRID: IMSR_JAX:000665
Mouse: C57BL/6J	The Jackson Laboratory	Cat# 000664; RRID: IMSR_JAX:000664
Mouse: C57BLKS/J	The Jackson Laboratory	Cat# 000662; RRID: IMSR_JAX:000662
Mouse: C57BR/cdJ	The Jackson Laboratory	Cat# 000667; RRID: IMSR_JAX:000667
Mouse: C57 L/J	The Jackson Laboratory	Cat# 000668; RRID: IMSR_JAX:000668
Mouse: C58/J	The Jackson Laboratory	Cat# 000668; RRID: IMSR_JAX:000668
Mouse: CBA/J	The Jackson Laboratory	Cat# 000656; RRID: IMSR_JAX:000656
Mouse: DBA/1J	The Jackson Laboratory	Cat# 000670; RRID: IMSR_JAX:000670
Mouse: DBA/2J	The Jackson Laboratory	Cat# 000671; RRID: IMSR_JAX:000671
Mouse: FVB/NJ	The Jackson Laboratory	Cat# 001800; RRID: IMSR_JAX:001800
Mouse: I/LnJ	The Jackson Laboratory	Cat# 000674; RRID: IMSR_JAX:000674
Mouse: KK/HiJ	The Jackson Laboratory	Cat# 02106; RRID: IMSR_JAX:02106
Mouse: LP/J	The Jackson Laboratory	Cat# 000676; RRID: IMSR_JAX:000676
Mouse: MA/MyJ	The Jackson Laboratory	Cat# 000677; RRID: IMSR_JAX:000677
Mouse: MRL/MpJ	The Jackson Laboratory	Cat# 000486; RRID: IMSR_JAX:000486
Mouse: NOD/ShiLtJ	The Jackson Laboratory	Cat# 001976; RRID: IMSR_JAX:001976
Mouse: NZB/BINJ	The Jackson Laboratory	Cat# 000684; RRID: IMSR_JAX:000684
Mouse: NZO/HlLtJ	The Jackson Laboratory	Cat# 02105; RRID: IMSR_JAX:02105
Mouse: NZW/LacJ	The Jackson Laboratory	Cat# 001058; RRID: IMSR_JAX:001058
Mouse: PL/J	The Jackson Laboratory	Cat# 000680; RRID: IMSR_JAX:000680
Mouse: SJL/J	The Jackson Laboratory	Cat# 000686; RRID: IMSR_JAX:000686
Mouse: SM/J	The Jackson Laboratory	Cat# 000687; RRID: IMSR_JAX:000687
Mouse: BXD1/TyJ	The Jackson Laboratory	Cat# 000036; RRID: IMSR_JAX:000036
Mouse: BXD2/TyJ	The Jackson Laboratory	Cat# 000075; RRID: IMSR_JAX:000075
Mouse: BXD6/TyJ	The Jackson Laboratory	Cat# 000007; RRID: IMSR_JAX:000007
Mouse: BXD9/TyJ	The Jackson Laboratory	Cat# 000105; RRID: IMSR_JAX:000105
Mouse: BXD11/TyJ	The Jackson Laboratory	Cat# 000012; RRID: IMSR_JAX:000012
Mouse: BXD13/TyJ	The Jackson Laboratory	Cat# 000040; RRID: IMSR_JAX:000040
Mouse: BXD14/TyJ	The Jackson Laboratory	Cat# 000329; RRID: IMSR_JAX:000329
Mouse: BXD15/TyJ	The Jackson Laboratory	Cat# 000095; RRID: IMSR_JAX:000095
Mouse: BXD16/TyJ	The Jackson Laboratory	Cat# 000013; RRID: IMSR_JAX:000013
Mouse: BXD18/TyJ	The Jackson Laboratory	Cat# 000015; RRID: IMSR_JAX:000015
Mouse: BXD19/TyJ	The Jackson Laboratory	Cat# 000010; RRID: IMSR_JAX:000010
Mouse: BXD21/TyJ	The Jackson Laboratory	Cat# 000077; RRID: IMSR_JAX:000077
Mouse: BXD27/TyJ	The Jackson Laboratory	Cat# 000041; RRID: IMSR_JAX:000041
Mouse: BXD28/TyJ	The Jackson Laboratory	Cat# 000047; RRID: IMSR_JAX:000047
Mouse: BXD29/TyJ	The Jackson Laboratory	Cat# 010981; RRID: IMSR_JAX:010981
Mouse: BXD31/TyJ	The Jackson Laboratory	Cat# 000083; RRID: IMSR_JAX:000083
Mouse: BXD32/TyJ	The Jackson Laboratory	Cat# 000078; RRID: IMSR_JAX:000078
Mouse: BXD33/TyJ	The Jackson Laboratory	Cat# 003222; RRID: IMSR_JAX:003222
Mouse: BXD34/TyJ	The Jackson Laboratory	Cat# 003223; RRID: IMSR_JAX:003223
Mouse: BXD38/TyJ	The Jackson Laboratory	Cat# 003227; RRID: IMSR_JAX:003227
Mouse: BXD39/TyJ	The Jackson Laboratory	Cat# 003228; RRID: IMSR_JAX:003228
Mouse: BXD40/TyJ	The Jackson Laboratory	Cat# 003229; RRID: IMSR_JAX:003229
Mouse: BXD42/TyJ	The Jackson Laboratory	Cat# 03230; RRID: IMSR_JAX:03230
Mouse: BXD43/RwwJ	The Jackson Laboratory	Cat# 07093; RRID: IMSR_JAX:07093
Mouse: BXD48a/RwwJ	The Jackson Laboratory	Cat# 007139; RRID: IMSR_JAX:007139
Mouse: BXD49/RwwJ	The Jackson Laboratory	Cat# 007098; RRID: IMSR_JAX:007098
Mouse: BXD50/RwwJ	The Jackson Laboratory	Cat# 007099; RRID: IMSR_JAX:007099
Mouse: BXD51/RwwJ	The Jackson Laboratory	Cat# 007100; RRID: IMSR_JAX:007100
Mouse: BXD55/RwwJ	The Jackson Laboratory	Cat# 007103; RRID: IMSR_JAX:007103
Mouse: BXD56/RwwJ	The Jackson Laboratory	Cat# 007104; RRID: IMSR_JAX:007104
Mouse: BXD60/RwwJ	The Jackson Laboratory	Cat# 007105; RRID: IMSR_JAX:007105
Mouse: BXD61/RwwJ	The Jackson Laboratory	Cat# 007106; RRID: IMSR_JAX:007106
Mouse: BXD62/RwwJ	The Jackson Laboratory	Cat# 007107; RRID: IMSR_JAX:007107
Mouse: BXD63/RwwJ	The Jackson Laboratory	Cat# 007108; RRID: IMSR_JAX:007108
Mouse: BXD65/RwwJ	The Jackson Laboratory	Cat# 007110; RRID: IMSR_JAX:007110
Mouse: BXD68/RwwJ	The Jackson Laboratory	Cat# 007113; RRID: IMSR_JAX:007113
Mouse: BXD69/RwwJ	The Jackson Laboratory	Cat# 007114; RRID: IMSR_JAX:007114
Mouse: BXD70/RwwJ	The Jackson Laboratory	Cat# 007115; RRID: IMSR_JAX:007115
Mouse: BXD71/RwwJ	The Jackson Laboratory	Cat# 007116; RRID: IMSR_JAX:007116
Mouse: BXD73a/RwwJ	The Jackson Laboratory	Cat# 007124; RRID: IMSR_JAX:007124
Mouse: BXD75/RwwJ	The Jackson Laboratory	Cat# 007119; RRID: IMSR_JAX:007119
Mouse: BXD77/RwwJ	The Jackson Laboratory	Cat# 007121; RRID: IMSR_JAX:007121
Mouse: BXD83/RwwJ	The Jackson Laboratory	Cat# 007126; RRID: IMSR_JAX:007126
Mouse: BXD84/RwwJ	The Jackson Laboratory	Cat# 007127; RRID: IMSR_JAX:007127
Mouse: BXD85/RwwJ	The Jackson Laboratory	Cat# 007128; RRID: IMSR_JAX:007128
Mouse: BXD86/RwwJ	The Jackson Laboratory	Cat# 007129; RRID: IMSR_JAX:007129
Mouse: BXD89/RwwJ	The Jackson Laboratory	Cat# 007132; RRID: IMSR_JAX:007132
Mouse: BXD90/RwwJ	The Jackson Laboratory	Cat# 007133; RRID: IMSR_JAX:007133
Mouse: BXD98/RwwJ	The Jackson Laboratory	Cat# 007141; RRID: IMSR_JAX:007141
Mouse: BXD99/RwwJ	The Jackson Laboratory	Cat# 007142; RRID: IMSR_JAX:007142
Mouse: BXD100/RwwJ	The Jackson Laboratory	Cat# 007143; RRID: IMSR_JAX:007143
Mouse: BXD102/RwwJ	The Jackson Laboratory	Cat# 007145; RRID: IMSR_JAX:007145
Software and algorithms		
eCAVIAR	Hormozdiari et al., 2016^64^	https://github.com/fhormoz/caviar
FaST-LMM	Lippert et al., 2011^27^	https://github.com/fastlmm/FaST-LMM
FOCUS	Mancuso et al., 2019^59^	https://github.com/bogdanlab/focus
FUSION	Gusev et al., 2016^58^	http://gusevlab.org/projects/fusion/
GMMAT	Chen et al., 2016^28^	https://cran.r-project.org/web/packages/GMMAT/index.html
heritability	Kruijer and White, 2019^99^	https://cran.r-project.org/web/packages/heritability/index.html
hisat2	Kim et al., 2019^100^	http://daehwankimlab.github.io/hisat2/download/
htseq-count	Anders et al., 2015^101^	https://github.com/htseq/htseq
lme4	Bates et al., 2015^26^	https://github.com/lme4/lme4
lme4qtl	Ziyatdinov et al., 2018^32^	https://github.com/variani/lme4qtl
LocusZoom	Pruim et al., 2010^57^	https://github.com/statgen/locuszoom-standalone
R	R Core Team^102^	https://www.R-project.org
STAR	Dobin and Gingeras, 2016^103^	https://github.com/alexdobin/STAR

## Highlights

- Cocaine use has a genetic component, but human analysis is difficult
- Genetically diverse mice are evaluated for cocaine self-administration
- Genome-wide and transcriptome-wide association studies identify 32 loci
- *Trpv2*, a known cannabinoid receptor, plays a key role in cocaine use
