# Supplementary material for: Genetic pathways regulating the longitudinal acquisition of cocaine self-administration in a panel of inbred and recombinant inbred mice
Source: Cell Rep. Author manuscript; Available in PMC 2023 Sep 27. (PMC10530068; doi:10.1016/j.celrep.2023.112856)
Supplement: 1 [file NIHMS1928238-supplement-1.pdf]

Cell Reports, Volume *42*

Supplemental information

Genetic pathways regulating the longitudinal acquisition of cocaine self-administration in a panel of inbred and recombinant inbred mice

Arshad H. Khan, Jared R. Bagley, Nathan LaPierre, Carlos Gonzalez-Figueroa, Tadeo C. Spencer, Mudra Choudhury, Xinshu Xiao, Eleazar Eskin, James D. Jentsch, and Desmond J. Smith

Genes regulated by brain region and sex

Significant regulation due to brain region or sex was identified for transcript abundance (brain region, 19,297 transcripts; sex, 822; false discovery rate, FDR < 0.05),[S1] spliceforms (brain region, 1,275 spliceforms; sex, 6) and RNA editing (brain region, 299 editing sites; sex, 0) (**Figures S4B**, **S4D**, **S4F** and **S4H**). Brain region had a larger number of regulated gene expression events than either cocaine (5,111 transcripts, 31 spliceforms, 2 editing sites; FDR < 0.05) or sex. Significant GO enrichments included cellular and primary metabolic process for brain region, and histone modification and protein methylation for sex (**Figures S4I-S4K**).

Of the 299 editing sites significantly regulated by brain region, 13 resulted in non-synonymous coding region changes, including *Cadps*, *Tmem63b*, *Unc80* and *Cyfip2* (**Figure S4H**).[S2–S5]

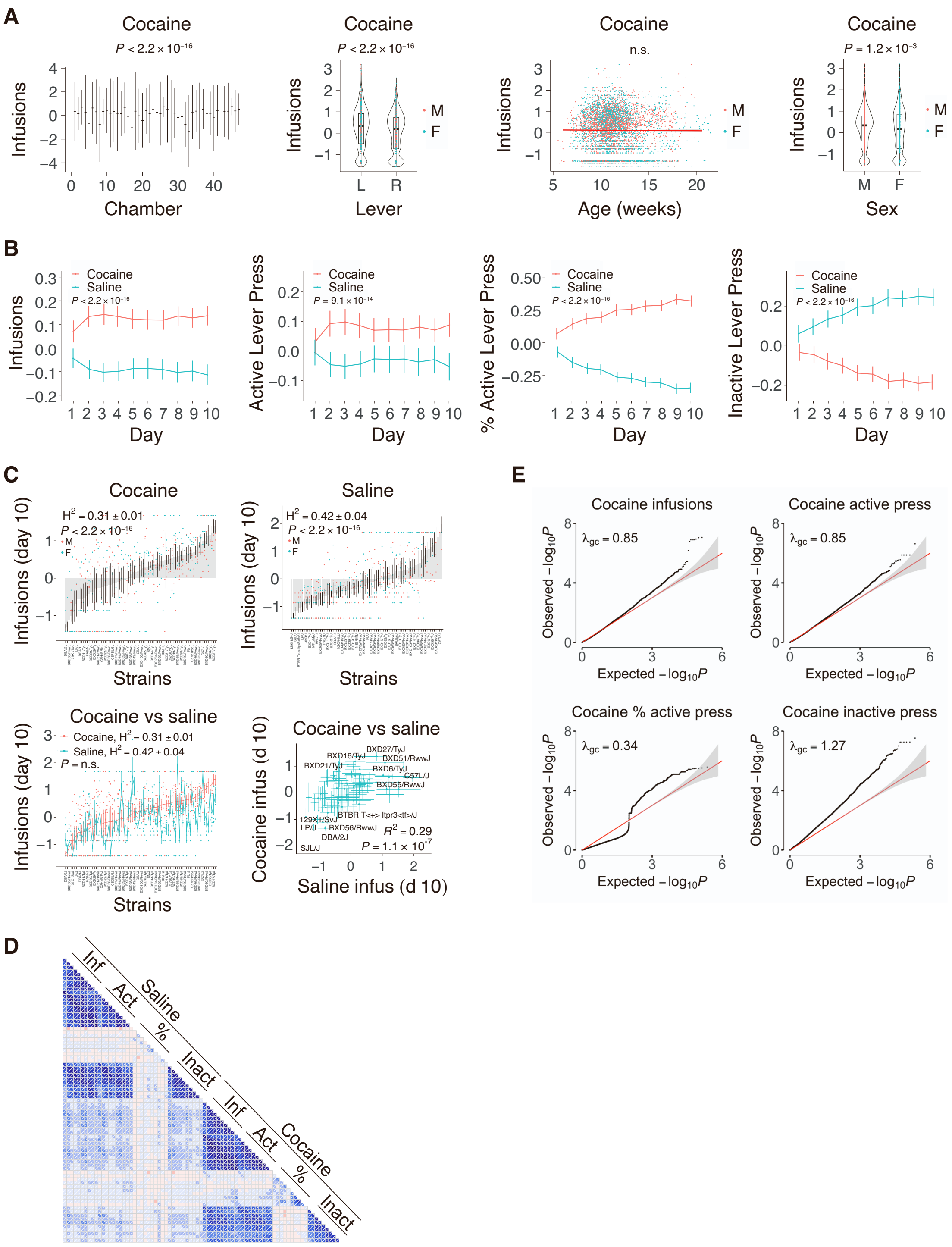

Figure S1. Behavioral covariates, H^2 and QQ (related to Figure 1). (**A**) Effects of covariates on normalized infusions. Testing chamber, median ± 1.75 × the interquartile range. (**B**) Cocaine vs saline IVSA. Normalized means ± s.e.m. *P*, cocaine vs saline. (**C**) Broad sense heritability (H^2) for day 10 infusions. Alternate strains labeled. *P*, H^2 vs null (individual plots); cocaine vs saline (joint plot). Scatterplot shows significant cocaine vs saline correlation. (**D**) Correlogram of IVSA GWAS $-\log_{10}P$ values. Rows and columns represent different testing days. (**E**) Quantile-quantile (QQ) plots for longitudinal cocaine genome scans. λ_{gc}, genomic control (inflation) factor.

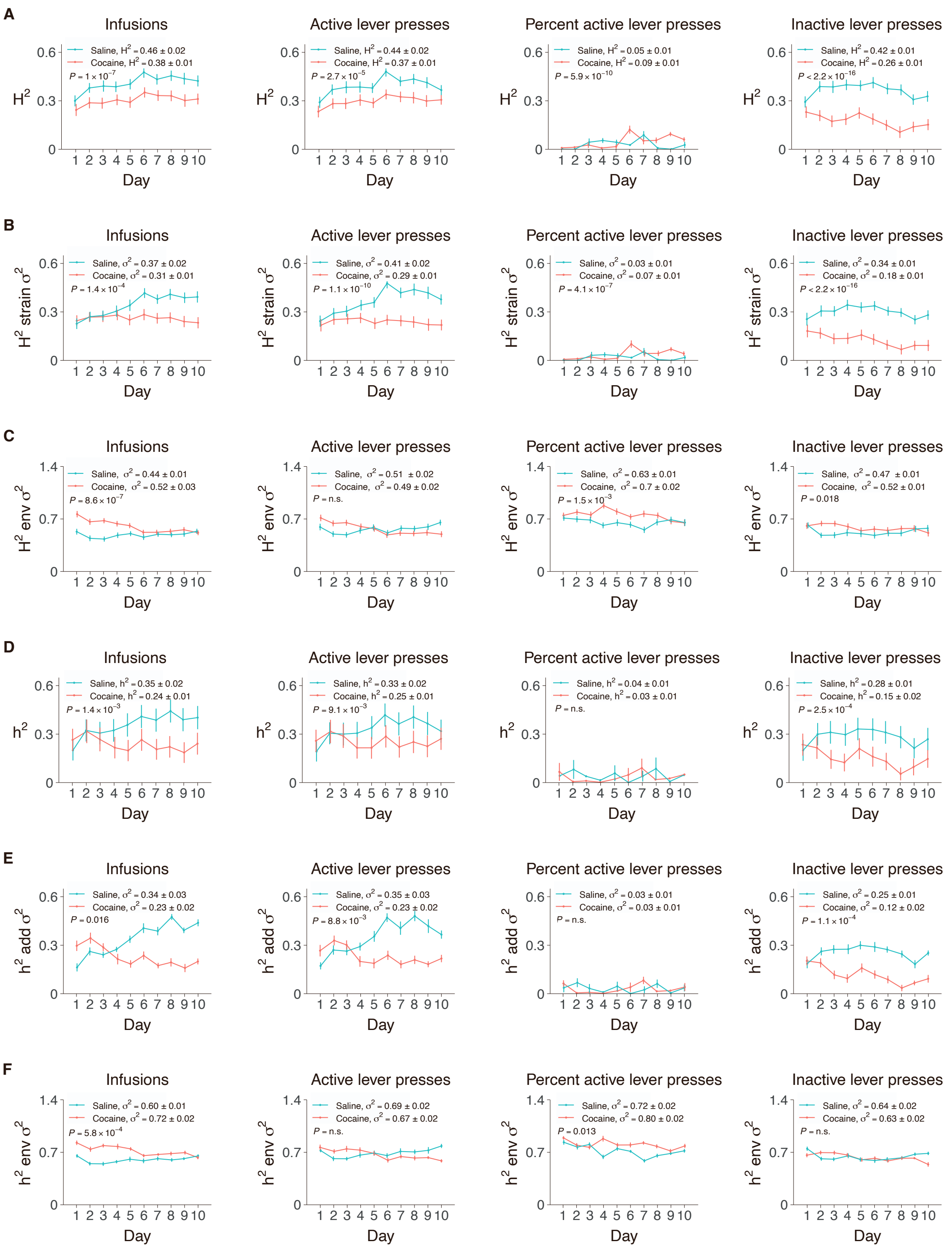

Figure S2. Heritability (related to Figure 1). (**A**) Broad sense heritability (H^2). (**B**) Strain variance (σ^2) from H^2. (**C**) Environmental variance (σ^2) from H^2. (**D**) Additive heritability (h^2). (**E**) Additive variance (σ^2) from h^2. (**F**) Environmental variance (σ^2) from h^2. *P* for saline vs. cocaine: H^2, sampling without replacement; h^2, linear mixed model with fixed effect of infusate and random effect of day. Means ± s.e.m.

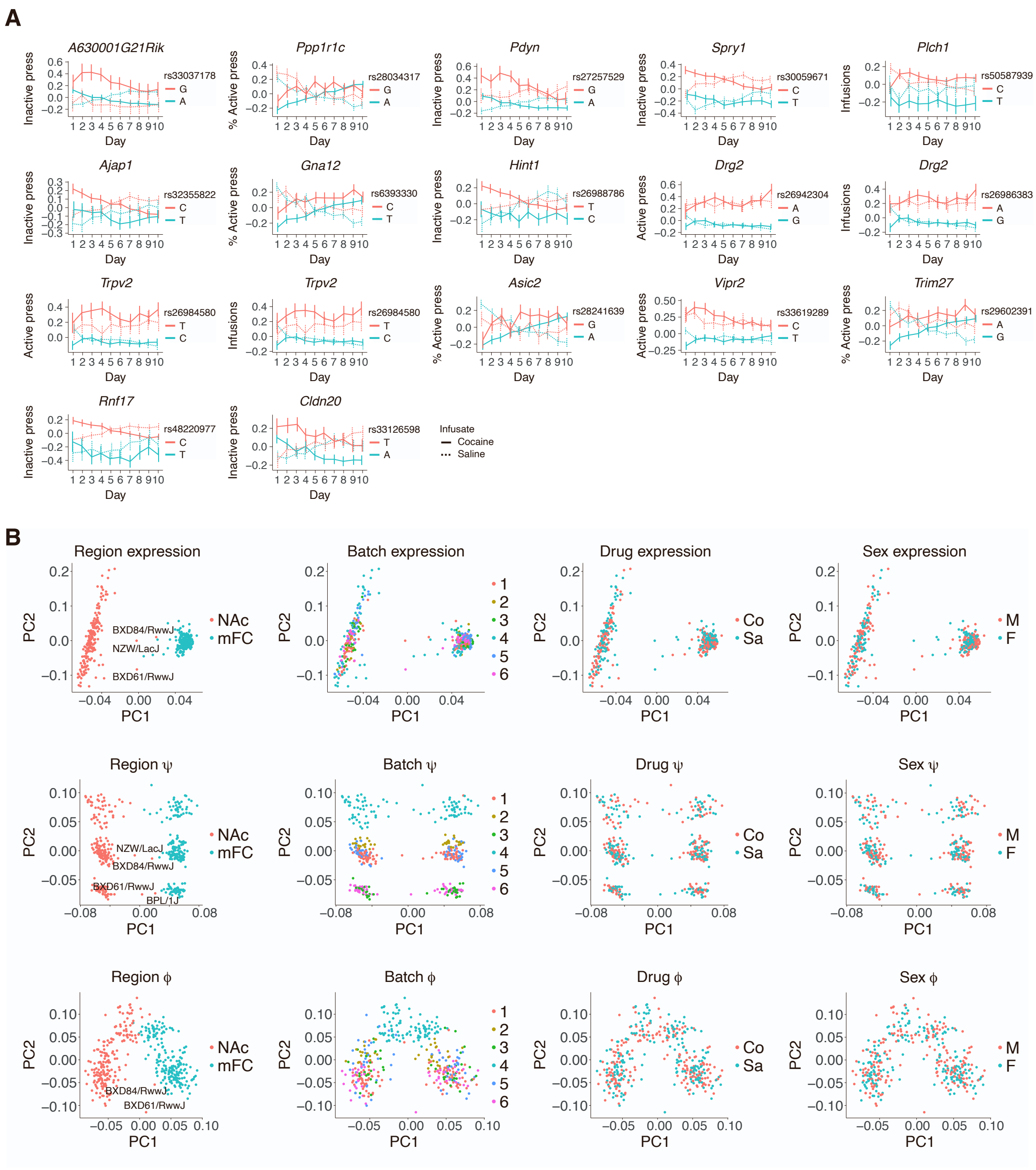

Figure S3. Allele effects on behavior and principal components analysis of RNA-seq (related to Figures 2, 3 and 5). (**A**) Allele effects on normalized cocaine IVSA from longitudinal genome scans. Loci in genomic order. Only one SNP was significant for saline using longitudinal model (rs30059671, inactive lever presses, *Spry1*, $P = 1.8 \times 10^{-6}$). (**B**) Principal components analysis (PCA) of RNA-seq. Variances: PC1, 0.26; PC2, 0.07 (expression); PC1, 0.03; PC2, 0.02 (splicing, ψ); PC1, 0.02; PC2, 0.02 (editing, ϕ). In plots colored by region, potentially misassigned samples labeled with strain names. All potentially misassigned samples have a correctly assigned sample of the same strain but opposite sex. Batch represents RNA-seq batches 1-6.

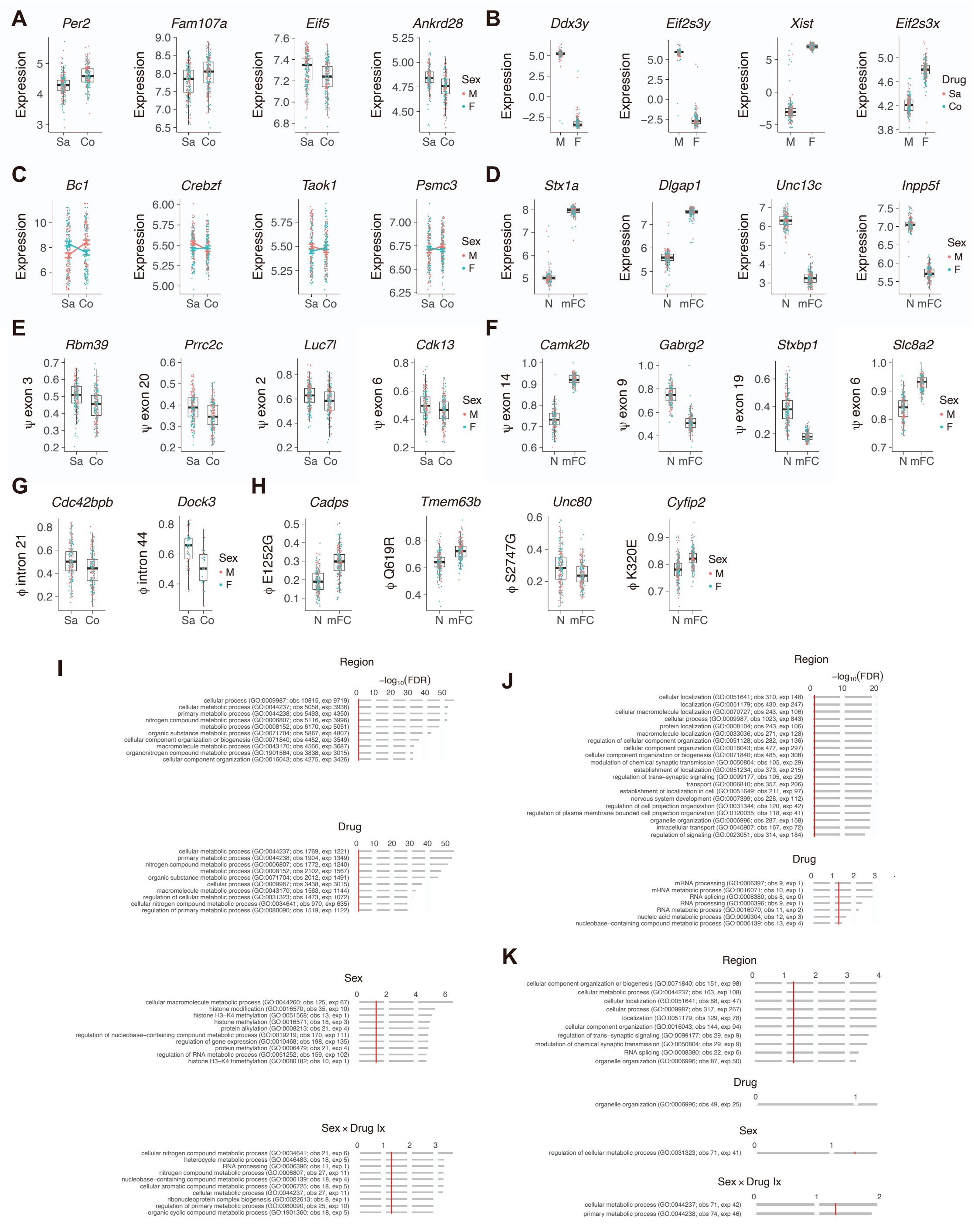

Figure S4. Regulation of gene expression (related to Figure 3). (**A**) Normalized transcript abundance regulated by infusate. (**B**) Sex. (**C**) Sex × infusate interaction. (**D**) Region. (**E**) Normalized percent spliced in (psi, ψ) regulated by infusate. (**F**) Region. (**G**) Normalized RNA editing ratio (ϕ) regulated by infusate. Both editing events are in intronic *Alu* elements, B1_Mus2 (*Cdc42bpb*: chr12: 111309987), B1_Mur1 (*Dock3*: chr9: 106905884). (**H**) Editing events regulated by region causing non-synonymous coding region changes. *Cadps*: NM_001042617: exon29: c.A3755G: p.E1252G; *Tmem63b*: NM_198167: exon20: c.A1856G: p.Q619R; *Unc80*: NM_001368824: exon54: c.A8239G: p.S2747G; *Cyfip2*: NM_133769: exon10: c.A958G: p.K320E. All FDRs < 0.05. (**I**) Gene ontology (GO) enrichment of transcripts. Biological process. Regulated genes with FDR < 0.05 chosen for analysis. Red lines, GO FDR = 0.05. obs, observed number of genes; exp, expected number of genes. (**J**) GO enrichment, splicing. Regulated exons with FDR < 0.05 chosen for analysis. (**K**) GO enrichment, RNA editing. Regulated editing sites with $P < 0.05$ chosen for analysis.

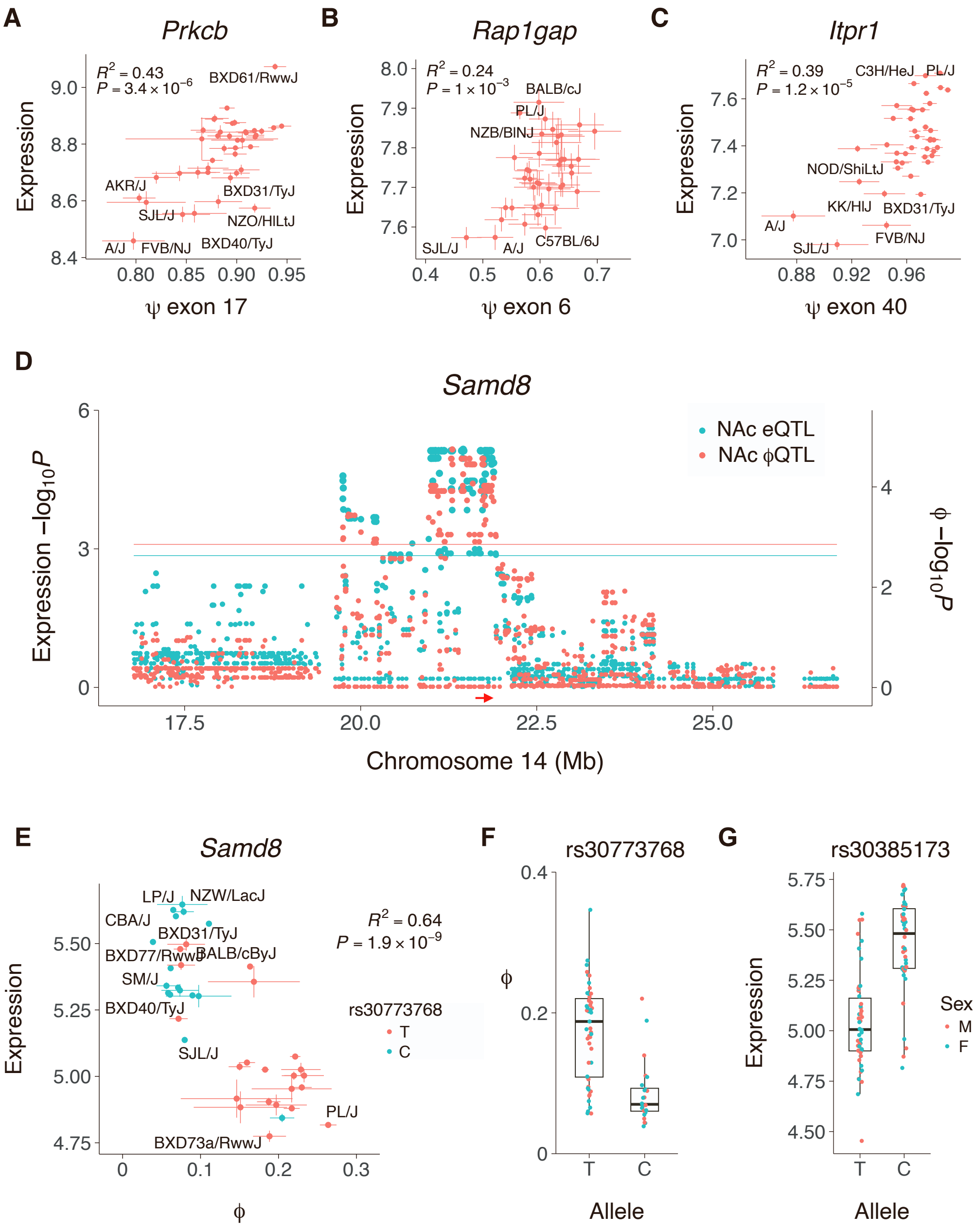

Figure S5. Splicing and RNA editing (related to Figures 3 and 4). (**A**) *Prkcb*, (**B**) *Rap1gap* and (**C**) *Itpr1* spliceforms affect RNA abundance. Normalized percent spliced in (ψ) and expression. Strain means ± s.e.m. R^2 and P values are strain averaged results, linear mixed model FDRs < 2.2×10^{-16}. (**D**) Coincident *Samd8 cis* editing QTL (ϕQTL) and *cis* expression QTL (eQTL) in NAc cocaine. Editing location at 21,797,711 bp on Chromosome 14 in B1_Mm *Alu* element in the 3' untranslated region of *Samd8*. Red arrow, location of *Samd8*. Red and blue horizontal lines, respective significance thresholds. (**E**) Allele T of rs30773768, the SNP most strongly correlated with *Samd8* editing, associated with higher *Samd8* editing and lower expression. Normalized strain means ± s.e.m. (**F**) Allele effect of rs30773768 on ϕ of *Samd8*. Individual samples shown. (**G**) Allele effect of rs30385173, the SNP most strongly correlated with *Samd8* expression. SNPs rs30385173 and rs30773768 are in linkage disequilibrium (D' = 1, R^2 = 0.94, $P < 2.2 \times 10^{-16}$).

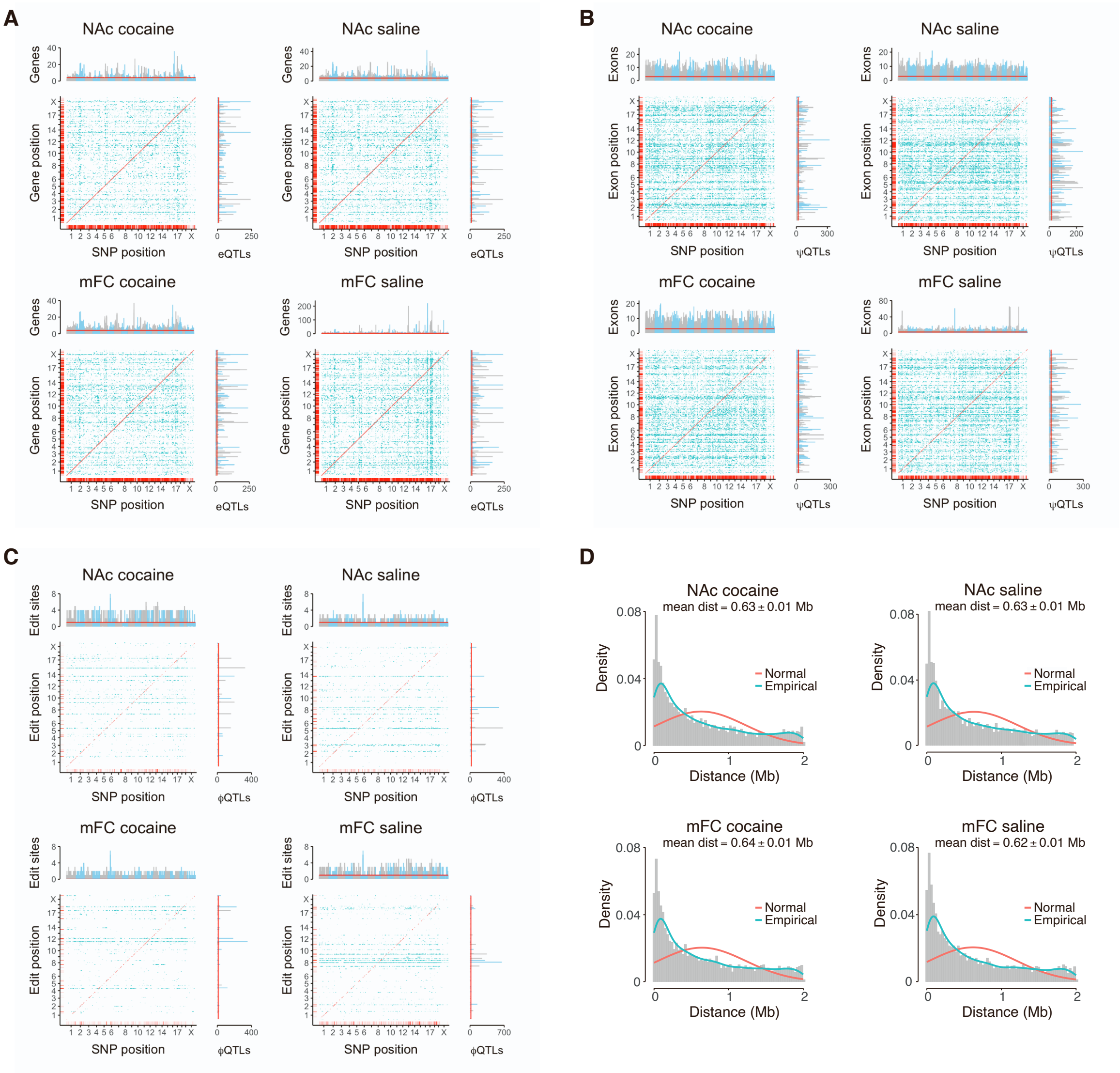

Figure S6. *Cis* and *trans* RNA quantitative trait loci (QTLs) (related to Figure 4). (**A**) Expression QTLs, eQTLs. NAc cocaine, *cis* (red) 4,469; *trans* (blue) 10,463. NAc saline, *cis* 4,699; *trans* 13,354. mFC cocaine, *cis* 5,152; *trans* 14,609. mFC saline, *cis* 5,057; *trans* 18,091. Marginal plots, red lines, FDR < 0.05 (Poisson). (**B**) Splicing QTLs, ψQTLs. NAc cocaine, *cis* 1,398; *trans* 25,140. NAc saline, *cis* 1,385; *trans* 26,276. mFC cocaine, *cis* 1,469; *trans* 25,532. mFC saline, *cis* 1,450; *trans* 25,028. (**C**) RNA editing QTLs, ϕQTLs. NAc cocaine, *cis* 290; *trans* 2,507; editing ascertainment rate, 36 ± 0.3% of samples. NAc saline, *cis* 284; *trans* 2,614; ascertainment, 36 ± 0.3%. mFC cocaine, *cis* 274; *trans* 1,880; ascertainment, 37 ± 0.3%. mFC saline, *cis* 240; *trans* 3,301; ascertainment, 37 ± 0.3%. (**D**) Distances between *cis* eQTLs and the corresponding genes. Red line, normal distribution; turquoise line, empirical distribution.

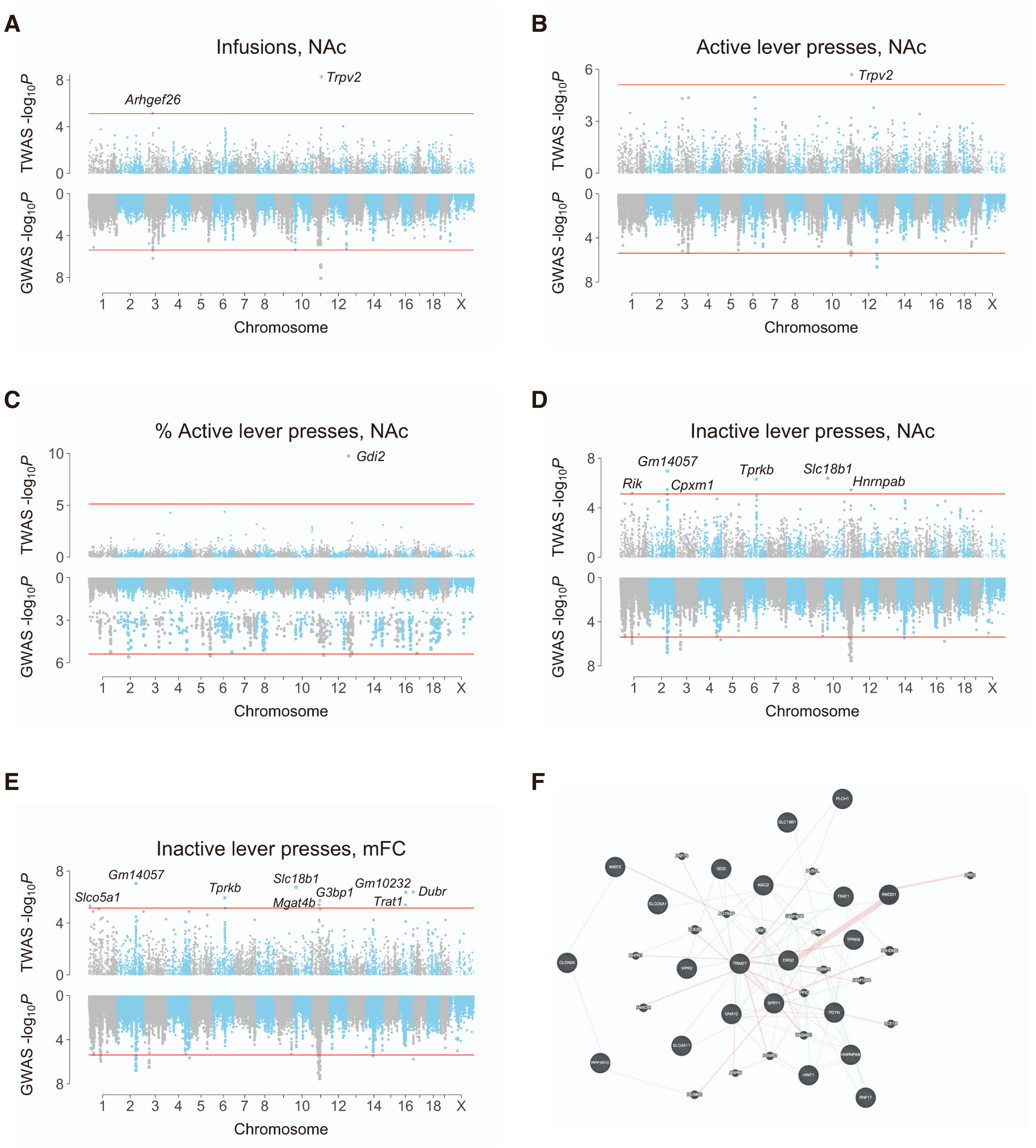

Figure S7. Transcriptome-wide association studies (TWAS) of cocaine IVSA (related to Figure 6). (**A**) Infusions, NAc. (**B**) Active lever presses, NAc. (**C**) Percent active lever presses, NAc. (**D**) Inactive lever presses, NAc. (**E**) Inactive lever presses, mFC. All TWAS use `FUSION` to analyze RNA-seq data from cocaine-exposed mice. (**F**) Interactions of candidate genes from longitudinal behavioral GWAS and TWAS identified using geneMANIA. Cross-hatched nodes, candidate genes. Non-crosshatched nodes, interacting genes. Pink, protein interactions; purple, co-expression; blue, co-localization; green, genetic interactions.

Table S2. Transcriptome-wide association studies for cocaine self-administration (related to Figure 6)

Gene	Chr	Start (bp)	Assay	Tissue[1]	FUSION *P*	FOCUS pip[2]	Behavioral locus[3]
Slco5a1	1	12,866,549	Inact	mFC	4.8e-06		
A630001G21Rik	1	85,717,083	Inact	NAc	6.7e-06		Inact
A630001G21Rik	1	85,717,083	Inact	mFC		0.91	Inact
Cpxm1	2	130,390,775	Inact	NAc	3.4e-06		
Slc4a11	2	130,684,113	Inact	NAc		0.82	
Gm14057	2	130,803,501	Inact	NAc	1.1e-07		
Gm14057	2	130,803,501	Inact	mFC	9.1e-08		
Arhgef26	3	62,338,344	Infus	NAc	7.0e-06		
Gna12	5	140,758,408	% Act Press	NAc		0.94	% Act Press
Tprkb	6	85,911,865	Inact	NAc	5.0e-07		
Tprkb	6	85,911,865	Inact	mFC	1.1e-06		
Slc18b1	10	23,796,986	Inact	NAc	4.3e-07		
Slc18b1	10	23,796,986	Inact	mFC	1.8e-07		
9930111J21Rik2	11	49,015,874	Inact	mFC		0.80	
Mgat4b	11	50,210,890	Inact	mFC	4.1e-06		
Hnrnpab	11	51,600,100	Inact	NAc	3.5e-06		
Gm12216	11	53,783,418	Inact	mFC		0.90	
G3bp1	11	55,469,685	Inact	mFC	1.8e-06	0.91	
Mief2	11	60,728,398	Infus	mFC		0.99	
Trpv2	11	62,574,486	Act Press	NAc	2.0e-06	0.86	Act Press
Trpv2	11	62,574,486	Infus	NAc	5.4e-09	1.00	Infus
Gdi2	13	3,538,063	% Act Press	NAc	1.8e-10		
Trat1	16	48,730,774	Inact	mFC	4.1e-06		
Dubr	16	50,719,294	Inact	mFC	4.3e-07		
Gm10232	17	3,044,014	Inact	mFC	4.1e-07		

[1] mFC, medial prefrontal cortex; NAc, nucleus accumbens; from cocaine-exposed mice.
[2] posterior inclusion probability.
[3] Loci from longitudinal GWAS

References

[S1.] Benjamini, Y., and Hochberg, Y. (1995). Controlling the false discovery rate: a practical and powerful approach to multiple testing. J R Stat Soc Series B Stat Methodol *57*, 289–300.

[S2.] Cuddleston, W.H., Li, J., Fan, X., Kozenkov, A., Lalli, M., Khalique, S., Dracheva, S., Mukamel, E.A., and Breen, M.S. (2022). Cellular and genetic drivers of RNA editing variation in the human brain. Nat Commun *13*, 2997. https://doi.org/10.1038/s41467-022-30531-0.

[S3.] Shumate, K.M., Tas, S.T., Kavalali, E.T., and Emeson, R.B. (2021). RNA editing-mediated regulation of calcium-dependent activator protein for secretion (CAPS1) localization and its impact on synaptic transmission. J. Neurochem. *158*, 182–196. https://doi.org/10.1111/jnc.15372.

[S4.] Tariq, A., Garncarz, W., Handl, C., Balik, A., Pusch, O., and Jantsch, M.F. (2013). RNA-interacting proteins act as site-specific repressors of ADAR2-mediated RNA editing and fluctuate upon neuronal stimulation. Nucleic Acids Research *41*, 2581–2593. https://doi.org/10.1093/nar/gks1353.

[S5.] Wu, D., Zang, Y.-Y., Shi, Y.-Y., Ye, C., Cai, W.-M., Tang, X.-H., Zhao, L., Liu, Y., Gan, Z., Chen, G., et al. (2020). Distant coupling between RNA editing and alternative splicing of the osmosensitive cation channel Tmem63b. Journal of Biological Chemistry *295*, 18199–18212. https://doi.org/10.1074/jbc.RA120.016049.
